# A Machine Learning Pipeline to Analyze Global Sentiment and Factors Influencing Retinoblastoma Treatment Hesitancy: Observational Infodemiology Study

**DOI:** 10.2196/73364

**Published:** 2026-08-11

**Authors:** Emily S Wong, Richard W Choy, Esther W Tang, Yuzhou Zhang, Xiu Juan Zhang, Linbin Zhou, Wai Kit Chu, Li Jia Chen, Clement C Tham, Chi Pui Pang, Jason C Yam

**Affiliations:** 1 Department of Ophthalmology and Visual Sciences, Chinese University of Hong Kong, 147K Argyle Street, Hong Kong, China (Hong Kong), 852 3943 5815; 2 Hong Kong Eye Hospital, Hong Kong, China (Hong Kong); 3 Department of Ophthalmology, Hong Kong Children’s Hospital, Hong Kong, China (Hong Kong); 4 Hong Kong Hub of Paediatric Excellence, Chinese University of Hong Kong, Hong Kong, China (Hong Kong); 5 Department of Ophthalmology, Li Ka Shing Faculty of Medicine, The University of Hong Kong, Hong Kong, China (Hong Kong); 6 Department of Ophthalmology and Visual Sciences, Prince of Wales Hospital, Hong Kong, China (Hong Kong)

**Keywords:** social media, retinoblastoma, ophthalmology, natural language processing, sentiment analysis, network analysis

## Abstract

**Background:**

The use of social media in cancer research, patient support, and information sharing has been well documented.

**Objective:**

Using retinoblastoma as a model, we use the information provided from Twitter (subsequently rebranded X) to understand patients’ treatment-seeking behavior and barriers, as well as investigate its application in research and epidemiology for rare diseases.

**Methods:**

Posts on retinoblastoma were extracted from Twitter. We trained BERT (Bidirectional Encoder Representations from Transformers) models to identify relevance and conducted sentiment analysis. The hierarchical Dirichlet process was trained to identify topics with BERTopic used as a sensitivity analysis. We enriched user profiles with OpenStreetMap-based geotagging and CoreNLP-based occupation tagging. Retinoblastoma outcomes were obtained from a systematic review and meta-analysis, which covered articles published between January 1, 1981, and October 8, 2021.

**Results:**

The dataset covered 2,382,511 posts from 797,870 Twitter users. Most of the information dissemination and discussion originated from North America and Western Europe. A lack of disease understanding and the need for more support and counseling remained the most significant barriers to receiving treatment worldwide, as reflected by both the intensity and number of posts. The number of new posts per year related to treatment barriers and enucleation hesitancy showed exponential growth after 2016 (β_log-linear_=0.957, *P*=.002). For the perceived barriers to treatment, sentiment was the strongest over time for worries over treatment failure (β_linear_=−0.003, *P*=.79, estimate_2022_=0.679). Posts with higher negative sentiment intensity related to enucleation were concentrated in Central and Southern America, Asia, and Africa. Stronger negative sentiment toward enucleation (*β*=−0.726, 95% CI −1.224 to −0.228) was associated with worse overall survival outcomes. The association between lower overall survival rates and enucleation hesitancy was observed in Asia (*β*=−1.518, 95% CI −2.602 to −0.434) and Africa (*β*=−0.812, 95% CI −1.412 to −0.021) in the subgroup analysis. The active participation of clinical staff (*β*=−0.105, 95% CI −0.186 to −0.024; *P*=.01) and academia (*β*=−0.116, 95% CI −0.208 to −0.024; *P*=.01) on retinoblastoma topics on Twitter correlated with lower enucleation hesitancy.

**Conclusions:**

Computational social media analysis can generate actionable insights for public health interventions for retinoblastoma. Negative sentiment toward enucleation is associated with poorer survival. The active participation of clinical staff and academia on Twitter is correlated with lower enucleation hesitancy. However, they remain underrepresented in social media discussions, suggesting a significant opportunity for greater engagement from stakeholders and targeted information dissemination to improve acceptance and outcomes in vulnerable zones.

## Introduction

Retinoblastoma is the most common intraocular childhood cancer, but treatment outcomes can be good especially with early management [1]. Although enucleation results in total vision loss and cosmetic concerns [2], it remains life-saving for patients at stage E under the Intraocular Classification of Retinoblastoma scheme [3,4]. With advancements in treatment techniques, especially globe salvage (GS) treatments, the overall survival (OS) and GS rates have increased over the past 4 decades globally [5,6]. Unfortunately, significant disparities persist between higher-income and lower-income countries, with some regions having significant survival gaps [7]. While access to health care services, late disease detection, and poor health care system performance has been recognized as critical barriers to improving survival rates [8,9], social and cultural factors can weaken disease understanding and acceptance of treatment options, resulting in late disease presentation and high default rates in certain regions [7,10-12]. A comprehensive understanding of the underlying barriers is necessary for targeted education and improvement in these regions [13-15].

Social media offers a significant bidirectional impact on public health care understanding [16]. On the one hand, it effectively drives public discourse and sentiments concerning health care interventions and modeling social beliefs. Posts on treatment access construct the perceived treatment barrier, and educational posts on disease management and progression without treatment shed light on perceived severity. By emphasizing the early signs and promoting early diagnosis, the prevention of global and life-threatening complications through early diagnosis underlies perceived benefits. Social media may influence health decisions as per the health belief model [17-21]. On the other hand, it provides a proxy for clinicians and researchers to systematically understand the health-seeking behavior and beliefs of patients [19,22-24]. Public sentiments may alter patients’ choices, and it would be strategic to use social media to disseminate accurate information that enhances health care outcomes [25-27]. Following the diffusion of innovation theory, key components of effective communications are determined by innovation, communication channels, time, and social systems. By forming highly connected, real-time focused networks around retinoblastoma and general pediatric eye care, social media usage entails both significant benefits and potential threats to the spread of legitimate medical information [28]. While this impact has been intensively studied in relation to COVID-19 vaccine hesitancy and misinformation, it is also relevant in noncommunicable diseases, including malignancies [16-21,23,24,26,27]. Existing applications of social media for oncology include patient support and patient-driven research. For example, patients with lung cancer have been using platforms such as Twitter (subsequently rebranded X) to offer and receive social and psychological support [18]. Twitter has also been used to evaluate patients’ adherence to treatment and recovery from urological diseases and prostate cancer [19]. The extensive use of social media has also been described for pediatric cancer and rare diseases, extending from emotional expression by survivors, information seeking by parents, and key information dissemination to the public. A summary of recent work focusing on the pediatric population has been provided in Table S1 in Multimedia Appendix 1.

To improve global awareness and acceptance of retinoblastoma treatment, it is essential to resolve underlying concerns and beliefs that hinder the understanding of retinoblastoma and its treatments. Perceived barriers to retinoblastoma treatment, such as cultural differences and gender inequality, are not well studied worldwide. We conducted this study to evaluate the use of social media’s power in characterizing global differences in retinoblastoma disease perception and to assess the impact on treatment choices and, subsequently, survival outcomes [29].

## Methods

### Search Strategy and Data Acquisition

We acquired data on discussions of retinoblastoma from Twitter. We accessed Twitter through its official API services [30]. We retrieved posts and subsequent discussions between January 1, 2011, and September 30, 2022. We searched for discussions relevant to retinoblastoma and imposed no limitations on language and origin. The search was conducted using the terms “retinoblastoma,” “enucleation,” or “eye cancer” and adapted to the search format for individual platforms. The search was conducted in the 6 official UN languages—Arabic, Chinese, English, French, Russian, and Spanish.

### Data Preprocessing and Filtering

We employed the DeepL translator service to translate the texts into English from their original language [31]. Manual validation was performed with 1000 sampled non-English posts to ensure accurate translation. The translation performance of the DeepL translator was further supported by a previous benchmarking study, demonstrating outstanding accuracy and outperforming other existing solutions [32]. We excluded irrelevant records, for example, those related to nonretinoblastoma eye malignancies and enucleation for nonretinoblastoma causes, using deep learning models. We fine-tuned a BERT (Bidirectional Encoder Representations from Transformers) model to filter texts from Twitter. The training (n=4000) and validation datasets (n=1000) were randomly sampled from the entire dataset and interpreted by ESW and RWC. Texts were labeled as relevant or irrelevant, and discrepancies were resolved by consensus. We fine-tuned the base BERT model on English texts using 10-fold cross-validation and validated on the holdout set [33,34].

### Topic Modeling

We performed topic modeling on the resultant relevant records stratified by platforms. Texts were cleaned by removing hyperlinks, hashtags, and user mentions and were further stemmed and lemmatized. Using the automated hierarchical Dirichlet process (HDP), we generated keywords that explained the topic distribution, which were sampled automatically from the input text word distribution [35]. Sensitivity analysis was performed using BERTopic, an embedding-based method. BERTopic uses embedding models to capture the semantic relationship for posts. Compared to HDP, it might be more resilient to short text with noise but requires higher computational power. On the contrary, HDP adopts a probabilistic Bayesian nonparametric approach. Apart from requiring less computational power, an essential strength of HDP is its ability to automatically infer the number of topics from a given dataset without prior assumptions. Hence, we compared the output from both models in this study.

Topics identified from both models were fused under several criteria. These included (1) topic keywords overlap, (2) semantic coherence and thematic redundancy, and (3) hierarchical proximity in the HDP model. For each topic, we computed the Jaccard similarity coefficient between the top 15 words for each topic pair, and those with a similarity score above 0.3 were highlighted for a manual review. In addition, topics with conceptual similarity were also reviewed manually, even if the similarity score was lower than the threshold, as semantic similarity may not be fully represented by token frequency similarity. Topic fusion was conducted independently by 2 authors and reviewed by a third author in case of discrepancies.

### Aspect-Based Sentiment Analysis

Similar to filtering posts, we fine-tuned a BERT model to classify posts into positive, neutral, and negative categories based on users’ acceptance of disease management. For example, posts representing the acceptance of treatment modalities while expressing sorrow for a diagnosis were labeled as positive [36]. The prediction probability was used to reflect the strength of emotions, that is, how strongly the post belonged to the respective category. We implemented 10-fold cross-validation and tested combinations of learning rate, batch size, and epoch. We calculated the strength of negative sentiment toward different treatment modalities for further analysis.

We fine-tuned a BERT model to classify posts by patient roles, that is, whether the post is posted by patients and their direct relatives or by general users. Similar to the above training approach, we labeled 1000 posts and fine-tuned the base BERT model using 10-fold cross-validation.

### Data Enrichment and Global Comparison

As only Twitter provided the detailed data on time, conversation details, and geolocation with a broad global user base among the platforms, we performed subsequent global comparisons using Twitter data only.

A fraction of posts was initially geotagged, that is, provided with the country or region of origin. We extracted their likely location for the other posts using the author’s user profile, with geotagging [37] and subsequent geocoding with the OpenStreetMap API [38]. The current approach has been validated by prior studies, showing high accuracy and reliable spatial granularity [39,40].

Users’ occupations were analyzed using their user profiles through the named entity recognition technique. Descriptions were processed using the default Stanford CoreNLP (version 4.5.1) model [41]. We manually labeled 2000 profiles to benchmark the extraction algorithm’s accuracy, sensitivity, and specificity. Subsequently, we manually processed the identified titles and aggregated them into different sectors, including clinical medicine, education and academia, media outlets and creative media, religion, politics, and public administration, and mapped them back to individual users.

### Network Analysis

Network analysis was conducted using Gephi [42]. After identifying isolated communities and nodes, we removed them to focus on the largest connected component of the network to investigate the functional component of the online discussion. In addition, this avoids complicating the calculation of path distance as disconnected nodes represent infinite path distance. We calculated network density, centrality measures—including eigenvector centrality—modularity, average path length, and other network properties for the discussion network and performed community detection. Eigenvector centrality in particular measures how influential a user is to the social network by accounting for both the number of connections and the number of important nodes that one is connected to. Therefore, we used eigenvector centrality to select those with high centrality scores for subsequent analysis [43].

### Clinical Data for Retinoblastoma

We acquired the OS rate and GS rate for retinoblastoma from our group’s previous systematic review and meta-analysis. This study analyzed clinical outcomes by country or region from 2010 to 2021 [7].

### Statistical Analysis

Variables used for global comparisons were first aggregated to the country level by taking averages. These included sentiment strength for different topics and clinical outcomes including OS and GS. We computed associations between the sentiment distribution and clinical outcomes (ie, OS and GS) using multivariable linear regression, adjusting for the Human Development Index, a statistical composite index covering socioeconomic development, health infrastructure and outcomes, and standard of living. The subgroup analysis was conducted by repeating multivariable linear regression with data for each region. Similarly, the associations between the participation rate of various professions on social media and the resultant discussion sentiments and intensity were also analyzed using multivariable linear regression. Linear model assumptions, including normality, homoscedasticity, independence, and linearity, were tested using the Shapiro-Wilk test, Breusch-Pagan test, Durbin-Watson test, and Rainbow test, respectively. We performed multiple testing adjustments with sequential Bonferroni correction in R (Bell Laboratories) to the reported statistics with a balanced focus on reducing the false discovery rate and preserving statistical power. The analysis was conducted in Python 3.9.0 and R 4.2.2 for the complete study. The complete data flow, detailed choice of models, and configurations from the above steps are shown in Figure S1 in Multimedia Appendix 2.

### Ethical Considerations

This study was approved by the Joint Chinese University of Hong Kong–New Territories East Cluster Clinical Research Ethics Committee (reference number 2025.559). This study involved only the secondary analysis of publicly available social media data, with no attempt to reidentify individuals. The extracted data were obtained via Twitter Academic API with official approval and stored in encrypted storage. Only aggregated data were reported.

## Results

After preprocessing, the dataset covered 2,382,511 posts from 797,870 Twitter users. Geotagging was successfully performed for 130,940 (16.4%) users. Per-fold cross-validation statistics are included in Table S2A and S2B in Multimedia Appendix 1. The BERT model for relevance classification achieved an accuracy, *F*_1_-score, and area under the curve (AUC) of 96.00% (95% CI 95.13‐96.87), 96.42% (95% CI 95.65‐97.19), and 96.22% (95% CI 94.25‐98.19), respectively (Table S2C in Multimedia Appendix 1). The high AUC score indicated high predictive power for the models to determine the correct outcome class. The entity extraction pipeline for recognizing user occupation descriptions from their profiles achieved an accuracy, sensitivity, specificity, and AUC score of 0.975, 0.967, 0.976, and 0.972, respectively, on the annotated test set. Extracted occupation tags were then grouped and annotated manually.

Globally, most retinoblastoma-related posts originated from North America and Europe, followed by South America, Australia, and India. Posts were scarce in Africa and were mainly concentrated in South Africa, Nigeria, and Kenya. The origins of posts are visualized in Figure S2 in Multimedia Appendix 2.

Topic modeling with HDP consistently identified contents from four major topics: (1) retinoblastoma awareness campaigns, (2) patient support, (3) presentation and diagnosis of retinoblastoma, and (4) treatment for retinoblastoma (Table S3 in Multimedia Appendix 1, Figure S3 in Multimedia Appendix 2). Topic modeling with BERTopic identified similar topic categories with a different set of keywords (Table S4 in Multimedia Appendix 1 and Figure S4 in Multimedia Appendix 2). About one-third of posts from Twitter focused on raising awareness for retinoblastoma, followed by retinoblastoma treatment (Figure S3 in Multimedia Appendix 2).

**Figure 1.**
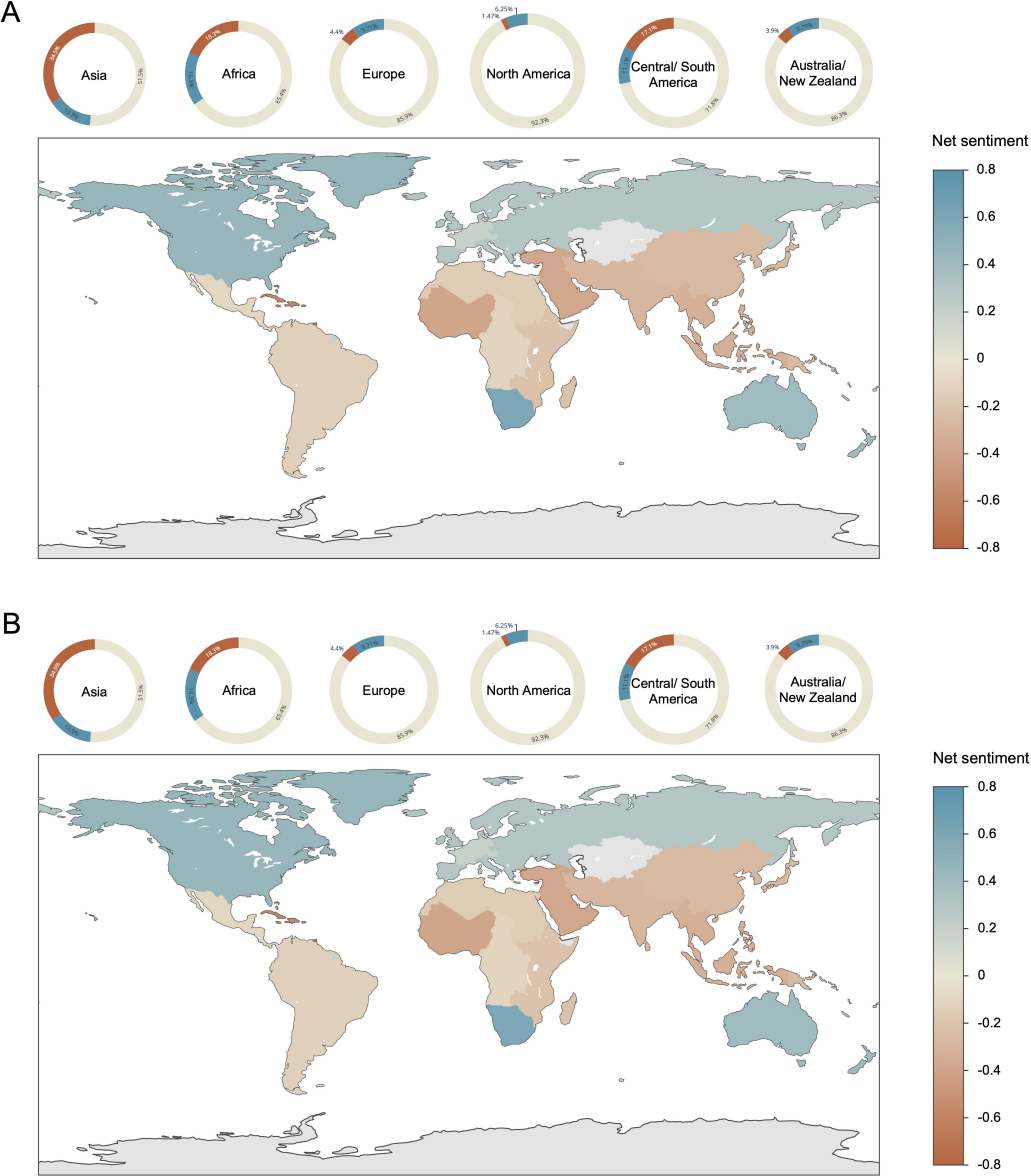
Twitter (subsequently rebranded “X”) post sentiment by location and topic only Twitter content was used for global sentiment comparison. (A) The global sentiment on patient support (such as patient group content, government funding, and insurances) and (B) treatment (such as modalities of treatment and the treatment outcomes). Sentiment analysis classified posts into positive (blue), neutral (cream white), and negative (red). The donut plots summarize the relative distribution of posts for each major region/continent, while the choropleth provides a more fine-grained visualization of the net sentiment by subregions.

The sentiment classification model achieved an AUC, *F*_1_-score, and accuracy of 0.861 (95% CI 0.844‐0.879), 0.708 (95% CI 0.677‐0.738), and 0.714 (95% CI 0.685‐0.742), respectively (Table S5 in Multimedia Appendix 1). For patient support, the strongest net negative sentiment was observed in Eastern Europe (−0.639) and Northern Africa (−0.458; Figure 1A). On the other hand, positive sentiment related to treatment was more prevalent in North America (+0.447), Europe (+0.186 to+0.296), Australia, and New Zealand (+0.398; Figure 1B), whereas negative sentiment toward treatment was more prevalent in Central (−0.095) and Southern America (−0.130), Southern (−0.284), and Western (−0.376) Asia (Figure 1B).

On multivariable linear regression with multiple testing adjustment, stronger negative sentiment toward enucleation was associated with worse OS outcomes (β=−0.726, 95% CI −1.224 to −0.228; *P*<.003; Table 1). Such an association was not observed for sentiment toward chemotherapy (β=−0.138, 95% CI −0.469 to 0.193; *P*=.67; Table 1). The association between survival rate and enucleation hesitancy was observed for Asia (β=−1.518, 95% CI −2.602 to −0.434; *P*=.007) and Africa (β=−0.812, 95% CI −1.412‐-0.021; *P*=.04) in the subgroup analysis (Table S6A in Multimedia Appendix 1). Tests on linear model assumptions were statistically insignificant, showing adherence to linear model assumptions (Table S6B in Multimedia Appendix 1).

**Table 1.** Association between negative post sentiment toward treatment and clinical outcomes (n=46)^a^.

Clinical outcome and treatment	β (95% CI, adjusted)	Adjusted *P* value	Adjusted *R*^2^
Overall survival			
Chemotherapy	−0.138 (−0.469 to 0.193)	.67	0.489
Enucleation	−0.726 (−1.224 to −0.228)	.003	0.599
Globe salvage			
Chemotherapy	−0.283 (−0.735 to 0.168)	.30	0.317
Enucleation	−0.362 (−1.102 to 0.378)	.52	0.316

a Linear regression was performed for the association between the strength of negative sentiment toward chemotherapy and enucleation and adverse clinical outcomes, such as poorer overall survival and lower globe salvage rate. Stronger negative sentiment toward enucleation (but not chemotherapy) was associated with worse overall survival outcomes. β=−0.726 (−1.224 to −0.228), *P*=.003.

**Figure 2.**
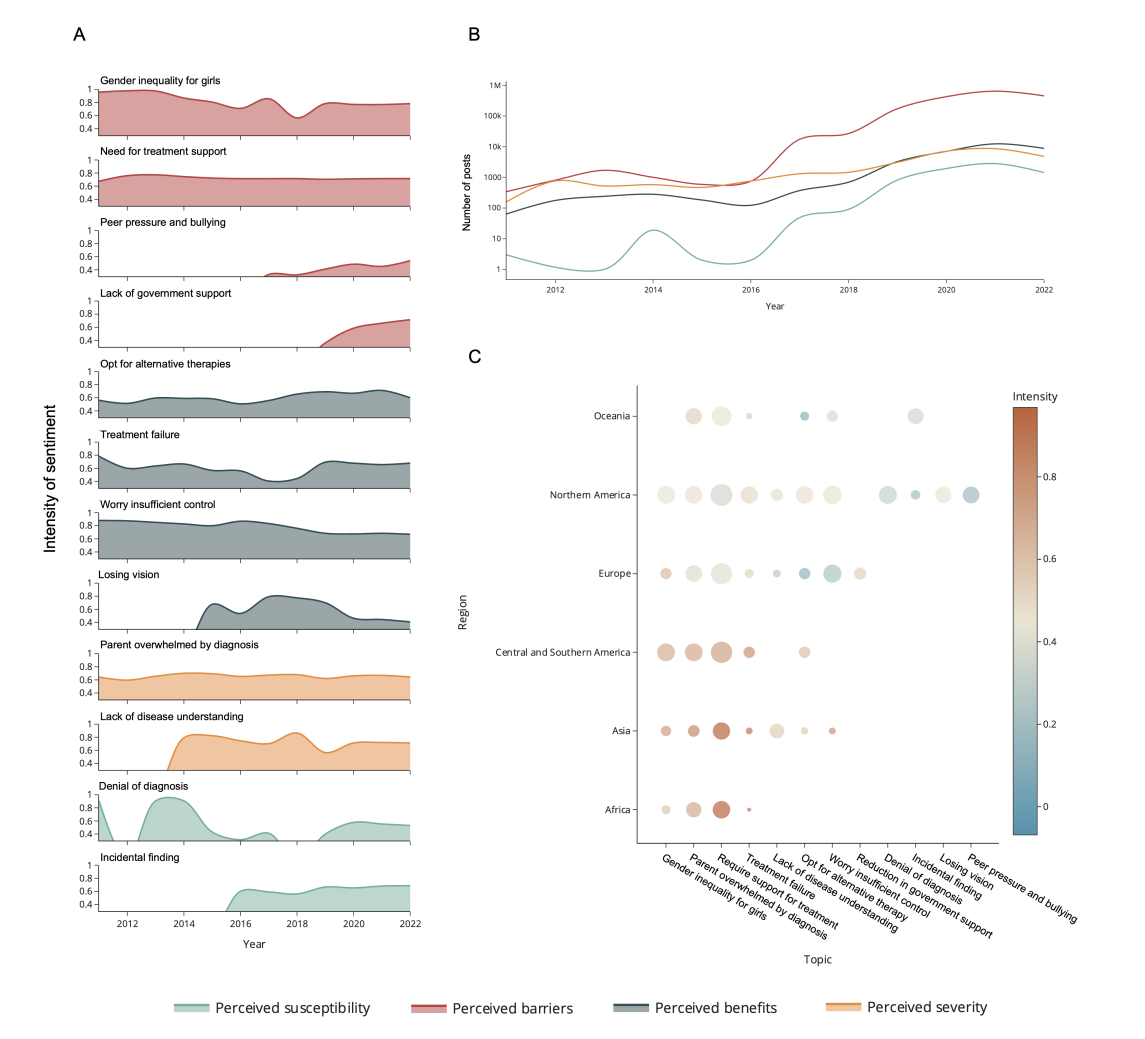
Topic distribution and trends of sentiment intensity related to treatment hesitancy. We extracted posts with negative sentiment for enucleation and performed topic modeling with the hierarchical Dirichlet process. We grouped the reasons into 12 topics under 4 major domains of the health belief model. The full range of identified reasons is included in Table S3 in Multimedia Appendix 1. (A) The time trend in the intensity of negative sentiment for each topic. (B) The number of posts per year in each domain over the past decade. The y-axis is in logarithmic for better visualization. (C) The bubble plot demonstrates the distribution of both relative topic frequency (size of bubble) and sentiment (color) for each topic in different locations. Overall, the need for support for treatment (eg, requesting financial support) is the most popular topic across different regions.

We further investigated the trends and patterns related to treatment barriers and enucleation hesitancy (Table S9 in Multimedia Appendix 1). Globally, the number of new posts per year related to treatment barriers and enucleation hesitancy remained steady until 2016 (β_linear_=148.2, *P*=.59), with exponential growth thereafter across all topics of the health belief model characterized by log-linear regression due to nonlinearity (β_log-linear_=0.957, *P*=.002; Figure 2). The growth occurred during a period of plateaued total user growth for Twitter [44]. For the perceived barriers to treatment, sentiment was strongest over treatment failure in 2022 (β_linear_=−0.003, *P*=.79, estimate_2022_=0.679), with no statistically significant linear trend over years. There was only moderate interest in seeking alternative therapies such as traditional Chinese medicine or herbal medicine (β_linear_=0.012, *P*=.02, estimate_2022_=0.599). There was a reduced sentiment intensity for worry about insufficient disease control (β_linear_=−0.021, *P*<.001, estimate_2022_=0.669) and losing vision (β_linear_=−0.090, *P*=.004, estimate_2022_=0.408), particularly after 2017. There was a lower sentiment intensity for denial of diagnosis after 2015 (estimate_2014_=0.906, estimate_2022_=0.530). Looking into the geographical distribution by topic, posts with higher negative sentiment intensity related to enucleation were concentrated in Central and Southern America, Asia, and Africa. The need for treatment support after diagnosis remained the most significant barrier in these regions, as reflected by both intensity and number of posts. This is followed by parents being overwhelmed by diagnosis and gender inequality (Figure 2). There were concerns over gender inequality for girls, but a declining sentiment intensity relating to gender was observed. There was an increasing sentiment trend for peer pressure and bullying (β_linear_=0.043, *P*=.006, estimate_2022_=0.783) and a persistent need for treatment support (β_linear_=−0.002, *P*=.34) from 2011 to 2022. An increasing trend of sentiment intensity for lack of government support was particularly seen since 2019 (β_linear_=0.114, *P*=.04, estimate_2022_=0.716), overlapping with the COVID-19 pandemic.

We also examined the association between the authors’ backgrounds and the intensity of negative sentiment in subsequent treatment-related discussions. General users accounted for the majority of users (83.87%) on the global network. Media outlets and creative media creators, politicians, and religious services accounted for 11.18%, 1.17%, and 0.12% of users, respectively. There was a relatively low representation of clinical medicine practitioners and educators or academia who accounted for only 1.80% and 1.86% of users, respectively. However, our analysis showed that posts from users with a background in clinical medicine (β=−0.105, *P*=.01) and education and academia (β=−0.116, *P*=.01) were associated with a lower intensity of negative sentiments in subsequent discussions (Table 2). Posts from media outlets and creative media creators were significantly associated with higher interaction quantity (eg, replies, reposts, and quotes) compared to the users with a medical background for treatment-related posts on Twitter (β=8.268, *P*=.004; Table 2). Although impactful, medical practitioners and educators were less effective in creating passive responses than media outlets and politicians. However, they were much more effective in creating active responses, particularly in the range of 10 to 1000 responses (Figure S5 in Multimedia Appendix 2). The geographical distribution of the global network for the top 10,000 users with specific occupations, based on eigenvector centrality, is presented in Figure S6 in Multimedia Appendix 2.

**Table 2.** Association between the occupation of authors and information dissemination^a^.

User types	Negative sentiment for treatment in subsequent discussions		Interaction quantity	
	β (95% CI)	*P* value	β (95% CI)	*P* value
Clinical medicine	−.105 (−0.186 to −0.024)	.01	−.792 (−7.865 to 6.282)	.83
Education and academia	−.116 (−0.208 to −0. 024)	.01	−.528 (−8.064 to 7.007)	.89
Media outlet and creative media	−.029 (−0.081 to 0.023)	.27	8.268 (2.650 to 13.887)	.004
Politics and public administration	−.016 (−0.107 to 0.075)	.73	.852 (−6.626 to 8.330)	.82
Religion	.101 (−0.029 to 0.232)	.13	−1.447 (−13.564 to 10.670)	.82

a We investigated the associations between the occupation of the author and (1) the intensity of negative sentiment for treatment and (2) the frequency of subsequent interactions in discussions using linear regression. Posts from users with a background in clinical medicine (β=−0.105, *P*=.01) and education and academia (β=−0.116, *P*=.01) were associated with a lower intensity of negative sentiments in subsequent discussions. Posts from media outlets and creative media were associated with a higher quantity of interaction (β=8.268, *P*=.004).

We further compared the sentiment related to retinoblastoma expressed by patient relatives and general users on social media. The classifier model for differentiating (1) patients and relatives and (2) other users achieved an AUC score of 0.813 (95% CI 0.793‐0.834; Table S7 in Multimedia Appendix 1). Statistically significant differences were observed across all domains after multiple testing adjustments, with the greatest differences noted in posts related to patient support (patient=−0.148, others=0.310, *P*<.001) and treatment (patient=−0.197, others=0.242, *P*<.001; Table S8 in Multimedia Appendix 1). The analysis suggests that targeted communication strategies may be beneficial for addressing the needs of these distinct groups.

Finally, for network analysis, multiple isolated communities were detected (n=22,875). After removing disconnected nodes and isolated communities, the analysis was performed for the remaining 796,546 nodes, revealing a very low directed density measure (estimate=3.27E-6), with average in-degree and out-degree both at 2.810, showing limited information exchange. Louvain modularity was estimated at 0.915, demonstrating a strong community structure. No significant quantity of bottleneck nodes was detected (ie, <10% of all users). The overall path length was measured at 3.99, which indicated a fragmented online community around retinoblastoma, underscoring the need for targeted outreach strategies to enhance information exchange and foster connections among isolated groups.

## Discussion

### Principal Results

To our knowledge, this is the first study to characterize the role of social media in global discussions of retinoblastoma. Our study provides a comprehensive picture of the real-world application of social media for public education and support of retinoblastoma, with discussions ranging from the promotion of retinoblastoma awareness campaigns to specific discussions on treatment and patient support groups. Being a rare disease, the wide base of direct user data provided by a global social media network like Twitter enables the exploration of subjective factors such as cultural perspectives, social viewpoints, the practice of alternative medicine, gender bias, etc, on retinoblastoma treatment acceptance and outcomes at a population-based level. This study extends the message from earlier publications on the impact of social media on pediatric cancer and rare diseases and further substantiates the context for its use in retinoblastoma. Building on the published approaches from previous social media studies, we provided an integrated pipeline for the systematic and structural analysis of social media platforms that can be readily used for various conditions [40,45-49]. Our study examined the association between discussion dynamics and the real-world clinical outcomes of retinoblastoma. Locations with generally negative sentiment toward retinoblastoma in parts of Asia and Africa correlated with zones of inferior survival rates, as identified in our earlier meta-analysis [7]. Negative sentiment and lack of education toward disease diagnosis and treatment contributed to delayed diagnosis and predisposed patients to the advanced clinical stage of retinoblastoma at presentation, often leading to limited treatment options and resulting in unsatisfactory GS and OS rates in these regions [5,6,9,11]. Other important factors such as health care accessibility also contributed to clinical outcomes across the world. The vast amount of user-generated content also allowed us to probe into the perceived barriers behind this negativity, which potentially resulted in delayed presentation or impeded discussions and understanding of retinoblastoma. While negative post sentiment does not directly cause poor clinical outcomes, it served as a proxy for understanding the underlying predisposing factors that inevitably aggravated the challenges to survival and surgical success.

We observed an association between a stronger negative sentiment toward enucleation and a lower OS rate for retinoblastoma patients worldwide. A large proportion of posts focused on the cost of treatment and rehabilitation related to vision loss. This concern was especially profound in resource-limited localities but also notably impacted resource-rich locations such as Western countries. Enucleation remains an important treatment modality for advanced retinoblastoma, with delayed treatment leading to metastasis and mortality. Alongside other clinical and socioeconomic factors that affect retinoblastoma outcomes, increased enucleation hesitancy may hence partly contribute to worse survival rates. While for the general population there might be well-established health care infrastructure to cover most patients’ needs, there were still unmet individual needs for patients, particularly for enucleation, which are associated with long-term visual impairment, socket complications, and required continual specialist support [50]. Various stakeholders, including governments and nongovernmental organizations, have been working rigorously to reinforce safety nets for retinoblastoma patients [51-53]. However, the current observation on Twitter may underpin a need to match patients with such resources to enhance use and alleviate patients’ burdens. The rising trend of the lack of patient support after diagnosis observed post-2019 overlaps with the COVID-19 pandemic, with frequent closures of treatment centers and delays in follow-up appointments. This might have affected the management of retinoblastoma patients. In the post–COVID-19 era, reversing such trends and perceptions would be paramount [54-56]. In a study in India, COVID-19–related full territory lockdowns were related to delayed and interrupted treatments, temporary or even permanent defaults on follow-ups, and mortality. Specifically, 60% of active cases prior to lockdown were shown to have worsened; 12 (5%) eyes were enucleated due to poor response to restarted treatment post default [54]. In a separate systematic review, care delivery was found to be particularly disrupted in lower- to middle-income countries compared to higher-income ones. Staff shortages, interrupted supply chains, and restricted accessibility to treatment facilities were among the most worrying situations [57]. Both online and offline interventions should hence be attempted to maximize the reach of such messages.

Other reasons for enucleation hesitancy, including the unpreparedness of parents and lack of disease understanding, should also be addressed in systemic and multilevel ways. Such causes may be due to inadequate counseling by health care workers upon diagnosis, denial of diagnosis, and sex stereotyping and discrimination against girls. Parent education is of paramount importance in retinoblastoma as parents are usually the ones who provide consent for treatment options. Clinical studies have shown the propensity for parents to develop anxiety and depression when their child is diagnosed with retinoblastoma, and support for their psychosocial needs is necessary for the best benefit of the patient [58]. On the other hand, parents should also be educated on the presentation, screening, and testing for retinoblastoma [59]. Greater knowledge of the disease has been associated with earlier diagnosis. On the contrary, parental shopping for alternative medical advice may result in disease progression and delay in diagnosis and treatment [60]. Gender stereotypes and sex bias, despite not being the main reason for treatment delays, still contribute to the constellation of concerns that aggravated enucleation hesitancy. Our prior study noted that sex ratios of retinoblastoma cases were significantly higher in some parts of Asia and Africa, despite no sex predilection for retinoblastoma. (1.30 vs 1.14, *P*=.04). Differences in gender health and mortality in South Asian countries have been identified previously, with inequality in access to care noted as a plausible explanation [10,61-63]. Discrimination and neglect of females for treatment due to sex-based referral bias have been reported in Nepal, Bangladesh, Pakistan, and China [10,64-69]. Our user-generated contents from Twitter show persistent yet declining intensity in sentiment related to sex inequality for girls over the years. Our data also demonstrated geospatial trends in the pattern of enucleation hesitancy, which allow for multilevel targeted health care interventions by trained personnel to improve acceptance in vulnerable zones and outcomes. Matching patient needs with existing resources can optimize resource usage and facilitate improvements in retinoblastoma outcomes on both regional and global scales.

Social media also proves to be an essential platform for health care workers to disseminate accurate information. In an editorial titled “Oncologists in social media—what are the limits?” from *The Lancet Oncology*, it was highlighted that despite their importance and effectiveness in information dissemination, social media sites may facilitate the dissemination of misinformation and pose a mental health burden. It is essential for health care professionals to use these sites effectively to benefit daily clinical practice [70]. A significant rise in the number of posts toward treatment hesitancy was observed in 2016. This corresponds to a major new development and renewed interest in GS therapies, including intra-arterial chemotherapy and intravitreal chemotherapy, the introduction of which greatly improved GS and OS rates, especially in developed countries. Unfortunately, our study showed that only a very small fraction of online information currently originates from health care workers. The interaction quantity of posts by health care workers is overshadowed by those from media or political outlets. Not only has this limited the potential benefits for resolving treatment hesitancy and introducing newer technology that could improve treatment outcomes, but it has also allowed the circulation of alternative information with variable levels of accuracy and reliability. Increased participation by local health care agencies, particularly in regions with higher treatment hesitancy, would be beneficial in generating locally appealing contents for treatment and disease acceptance. The reach can be further improved with cooperation and assistance from media outlets, creative media creators, government agencies, and nongovernmental organizations. Of note is that health advocacy on social media sites may lead to the establishment of doctor-patient relationships and carry duty-of-care concerns. We suggest that rather than providing consultation and advice on a case-by-case basis on social media platforms, agencies, whether government or nongovernment, should standardize critical information based on proven facts or the latest research about the disease that can be conveniently accessible to medical and patient support groups. The goals would be to establish factful discussions online and promote awareness of retinoblastoma. Training sessions could be provided to interested health care professionals to improve content delivery and communication strategies specific to social media platforms and techniques for mitigating misinformation as well as tackling legal and ethical concerns. Public health agencies shall also increase coverage and research in social media spaces and provide timely action if misinformation arises while maintaining high levels of disease awareness and health literacy. With the low latency and cross-border nature of social media sites, verified and translated content can be developed to target vulnerable regions identified via research, together with surveys for pinpointing patients in need. This would be particularly important for localities with fragmented health care systems and limited resources. Alongside existing international collaborations, this could provide a high-level intervention to improve retinoblastoma outcomes.

### Limitations

There are limitations to this study. First, although we have analyzed a large volume of social media posts and correlated them with global data from a large cohort, the ecological study approach restricted the interpretation of results to the population level. It is important to note that the identified relationships represent associations and should not be interpreted as direct causative effects. Second, due to privacy and ethical concerns, we were not able to individually verify the authenticity and background of user profiles for sentiment analysis and progression, limiting the generalizability of sentiment analysis and its correlation with underlying disease status and treatment acceptance. Third, due to local variation in the popularity of social media platforms and the lack of publicly available data for platforms such as Facebook, Weibo, and Line, we were not able to exhaust all the information online. It is essential to compare results from multiple platforms and incorporate the analysis of popular local platforms to integrate findings in this study into local practice. Moreover, this study included posts in 6 official UN languages. This might, however, introduce selection bias against populations in Asia and South America where the official languages were not covered. The results may not fully represent the general population in those locations and may be skewed toward English-speaking or Western-educated populations in these localities. Finally, the extracted data were in multiple languages, and translation was necessary for further comparative analysis. Although we used a validated translation tool for processing, there might still be potential nuances in the cultural context or linguistic subtleties lost in translation, affecting the accuracy of sentiment analysis. Future studies may collaborate with popular social media sites, following the proven importance of social media in disease management, to provide a more comprehensive analysis of health information distribution online. Validation studies may also check the accuracy of profiles and contents mentioned by users online. Moreover, a slight time discrepancy existed between the sentiment data and the clinical data. Sentiment data were collected in September 2022, with clinical outcomes collected only up to October 2021. This short mismatch was noted and may affect the reported association if clinical outcomes from October 2021 to September 2022, that is, the period without clinical outcomes report, were notably different from earlier periods. There may be a possibility of noncausal noise in the reported association accordingly. Furthermore, despite our efforts to include posts from global users and cover parts of Africa and Asia, ecological fallacies may persist. In particular, Twitter users in these regions may represent a socioeconomically distinct population, including those living in urban areas or those who are socioeconomically privileged. These users may not fully represent the general population reflected in the clinical survival data, resulting in ecological fallacy or skewed representation. Due to privacy settings and the inherent nature of social networking sites, geotagging was only successful for 16.4% of identified users. There might hence be underrepresentation or biased association estimates for certain regions. Ecological fallacy may also arise as associations were computed on country-level aggregates and may not represent associations at the individual level. For subgroup analysis, since there are a limited number of countries in each subset, the multivariable analysis with adjustment for the development index may introduce severe overfitting. The results only aimed to supplement the global association and to reflect potential regional differences with additional caution regarding the differences in the development level. The association should hence be interpreted with caution. In addition, despite the significant associations between treatment sentiment and clinical outcomes, the adjusted R-squared values range roughly between 0.316 and 0.599. The sentiment hence only partially explained the variance in clinical outcomes, and other contributing factors should not be overlooked. Despite the potential power of social media sites in providing timely global insights for disease conditions, the ethical implications should not be overlooked. Apart from user privacy, users from regions with restricted social media or political protections may be underrepresented. Policy decisions that solely rest on social media insights may hence paradoxically aggravate such inequity.

### Conclusion

Social media is extensively used for retinoblastoma education and patient support, particularly in North America and Western Europe. The improved understanding of parents and patients with retinoblastoma, as reflected by positive sentiment toward the disease and its treatment, and more engagement from clinicians, was associated with better OS and reduced enucleation hesitancy. Demonstrating geospatial trends in enucleation hesitancy allows for targeted information dissemination by health care workers to improve acceptance in vulnerable regions and reduce barriers to receiving timely treatment. Matching patient needs with existing resources can optimize resource usage and facilitate improvements in retinoblastoma outcomes on a regional and global scale.

## Supplementary material

10.2196/73364 Multimedia Appendix 1 Summary of existing work on social media and pediatric cancer and rare disease; overall cross-validation statistics for BERT model for text relevance classification, text sentiment classification, and patient role classification; topics identified by BERTopic; subgroup analysis and test for assumptions for multivariable regression model; sentiment comparison by patient role and topic; keywords identified by hierarchical Dirichlet process on general topic modeling and reasons of enucleation hesitancy.

10.2196/73364 Multimedia Appendix 2 Dataflow for analysis; global distribution of Twitter tweets; topic distribution for Twitter; BERTopic topic modeling; engagement statistics by author occupation; network plot global network of retinoblastoma information flow on Twitter.

## Abbreviations

AUC: area under the curve
BERT: Bidirectional Encoder Representations from Transformers
GS: globe salvage
HDP: hierarchical Dirichlet process
OS: overall survival

## References

[1] Delhiwala KS, Vadakkal IP, Mulay K, Khetan V, Wick MR (2016). Retinoblastoma: an update. Semin Diagn Pathol.

[2] Shields CL, Shields JA (1999). Recent developments in the management of retinoblastoma. J Pediatr Ophthalmol Strabismus.

[3] Linn Murphree A (2005). Intraocular retinoblastoma: the case for a new group classification. Ophthalmol Clin North Am.

[4] Shields CL, Mashayekhi A, Au AK (2006). The International Classification of Retinoblastoma predicts chemoreduction success. Ophthalmology.

[5] Ancona-Lezama D, Dalvin LA, Shields CL (2020). Modern treatment of retinoblastoma: a 2020 review. Indian J Ophthalmol.

[6] Fabian ID, Onadim Z, Karaa E (2018). The management of retinoblastoma. Oncogene.

[7] Wong ES, Choy RW, Zhang Y (2022). Global retinoblastoma survival and globe preservation: a systematic review and meta-analysis of associations with socioeconomic and health-care factors. Lancet Glob Health.

[8] Fabian ID, Abdallah E, Abdullahi SU, Global Retinoblastoma Study Group (2020). Global retinoblastoma presentation and analysis by national income level. JAMA Oncol.

[9] Canturk S, Qaddoumi I, Khetan V (2010). Survival of retinoblastoma in less-developed countries impact of socioeconomic and health-related indicators. Br J Ophthalmol.

[10] Fabian ID, Khetan V, Stacey AW (2022). Sex, gender, and retinoblastoma: analysis of 4351 patients from 153 countries. Eye (Lond).

[11] Fabian ID, Abdallah E, Abdullahi SU, Global Retinoblastoma Study Group (2022). The Global Retinoblastoma Outcome Study: a prospective, cluster-based analysis of 4064 patients from 149 countries. Lancet Glob Health.

[12] Utuk EOE, Ikpeme EE (2015). Childhood cancers in a referral hospital in south-south Nigeria: a review of the spectrum and outcome of treatment. Pan Afr Med J.

[13] Al-Nawaiseh I, Ghanem AQ, Yousef YA (2017). Familial retinoblastoma: raised awareness improves early diagnosis and outcome. J Ophthalmol.

[14] Yousef YA, AlNawaiseh T, AlJabari R, Muhsen S, Al-Nawaiseh I (2019). Retinoblastoma awareness among first contact physicians in Jordan. Ophthalmic Genet.

[15] Naser AY, Alrawashdeh HM, Alwafi H (2021). Knowledge and awareness of the general population and healthcare providers about retinoblastoma: it is time to know the glow. Int J Clin Pract.

[16] Fuentes A, Peterson JV (2021). Social media and public perception as core aspect of public health: the cautionary case of @realdonaldtrump and COVID-19. PLoS ONE.

[17] Suarez-Lledo V, Alvarez-Galvez J (2021). Prevalence of health misinformation on social media: systematic review. J Med Internet Res.

[18] Taylor J, Pagliari C (2019). The social dynamics of lung cancer talk on Twitter, Facebook and Macmillan.org.uk. NPJ Digit Med.

[19] Loeb S, Katz MS, Langford A, Byrne N, Ciprut S (2018). Prostate cancer and social media. Nat Rev Urol.

[20] Katz MS, Utengen A, Anderson PF (2016). Disease-specific hashtags for online communication about cancer care. JAMA Oncol.

[21] Loeb S, Catto J, Kutikov A (2014). Social media offers unprecedented opportunities for vibrant exchange of professional ideas across continents. Eur Urol.

[22] Clarke C, Smith E, Khan M, Al-Mohtaseb Z (2018). Social media and ophthalmology: perspectives of patients and ophthalmologists. J Med Syst.

[23] Usher K, Durkin J, Martin S (2021). Public sentiment and discourse on domestic violence during the COVID-19 pandemic in Australia: analysis of social media posts. J Med Internet Res.

[24] Cohen D, Allen TC, Balci S (2017). #InSituPathologists: how the #USCAP2015 meeting went viral on Twitter and founded the social media movement for the United States and Canadian Academy of Pathology. Mod Pathol.

[25] Chan KL, Chen M (2019). Effects of social media and mobile health apps on pregnancy care: meta-analysis. JMIR Mhealth Uhealth.

[26] Kim SH, Utz S (2019). Effectiveness of a social media–based, health literacy–sensitive diabetes self‐management intervention: a randomized controlled trial. J Nurs Scholarsh.

[27] Moorhead SA, Hazlett DE, Harrison L, Carroll JK, Irwin A, Hoving C (2013). A new dimension of health care: systematic review of the uses, benefits, and limitations of social media for health communication. J Med Internet Res.

[28] Spann B, Mead E, Maleki M, Agarwal N, Williams T (2022). Applying diffusion of innovations theory to social networks to understand the stages of adoption in connective action campaigns. Online Soc Net Media.

[29] Rodriguez-Galindo C, Wilson MW, Chantada G (2008). Retinoblastoma: one world, one vision. Pediatrics.

[30] X API. X Developer Platform.

[31] DeepL translate. DeepL.

[32] Linlin L (2024). Artificial intelligence translator DeepL translation quality control. Procedia Comput Sci.

[33] Cui Y, Che W, Liu T, Qin B, Wang S, Hu G Revisiting pre-trained models for Chinese natural language processing.

[34] Devlin J, Chang MW, Lee K, Toutanova K BERT: pre-training of deep bidirectional transformers for language understanding. https://aclanthology.org/N19-1423.pdf.

[35] Teh YW, Jordan MI, Beal MJ, Blei DM (2006). Hierarchical Dirichlet processes. J Am Stat Assoc.

[36] Barbieri F, Camacho-Collados J, Espinosa Anke L, Neves L TweetEval: unified benchmark and comparative evaluation for tweet classification.

[37] Locationtagger 0.0.1. PyPI.

[38] Curran K, Crumlish J, Fisher G (2012). OpenStreetMap. Int J Inter Commun Syst Technol.

[39] Kilic B, Hacar M, Gülgen F (2023). Effects of reverse geocoding on OpenStreetMap tag quality assessment. Trans GIS.

[40] Serere HN, Resch B, Havas CR, Petutschnig A (2021). Extracting and geocoding locations in social media posts: a comparative analysis. GI_Forum.

[41] Manning C, Surdeanu M, Bauer J, Finkel J, Bethard S, McClosky D The stanford corenlp natural language processing toolkit.

[42] Bastian M, Heymann S, Jacomy M (2009). Gephi: an open source software for exploring and manipulating networks. Proc Int AAAI Conf Web Soc Media.

[43] Bihari A, Pandia MK Eigenvector centrality and its application in research professionals’ relationship network.

[44] Has Twitter reached its natural growth limit?. Statista.

[45] Kilcoyne S, Overton S, Brockbank S (2024). Social media and website use: the experiences of parents and carers accessing care at the oxford craniofacial unit. J Craniofac Surg.

[46] Lau N, Zhao X, O’Daffer A, Weissman H, Barton K (2024). Pediatric cancer communication on Twitter: natural language processing and qualitative content analysis. JMIR Cancer.

[47] Johnson A, Widger K (2025). Harnessing social media to develop conceptual domains of quality of life for adolescents with advanced cancer. Psychooncology.

[48] Oluwalade TI, Ahmadi H, Huo L, Sharpe R, Zhou SM (2025). Comorbidities and emotions - unpacking the sentiments of pediatric patients with multiple long-term conditions through social media feedback: a large language model-driven study. J Affect Disord.

[49] Honda N, Funakoshi Y, Matuishi Y, Morifuji K, Tanabe K (2025). Adolescent childhood cancer survivors talking about cancer: a socioecological perspective. Asia Pac J Oncol Nurs.

[50] Gibbs D, Reynolds L, Shea Yates T (2022). Understanding the experiences of living with an artificial eye in children with retinoblastoma—perspectives of children and their parents. J Pediatr Hematol Oncol Nurs.

[51] The 2020 campaign: train 20 specialists in 20 countries to treat childhood eye cancer. New York Eye Cancer Center.

[52] Torres-Netto EA, Gabel-Obermaier C, Gabel P (2021). Twenty years of International Council of Ophthalmology fellowships: description of the programme and the impact on more than 1100 awardees. Br J Ophthalmol.

[53] Hill JA, Kimani K, White A (2016). Achieving optimal cancer outcomes in East Africa through multidisciplinary partnership: a case study of the Kenyan National Retinoblastoma Strategy group. Global Health.

[54] Bansal R, Aishwarya A, Rao R (2021). Impact of COVID-19 nationwide lockdown on retinoblastoma treatment and outcome: a study of 476 eyes of 326 children. Indian J Ophthalmol.

[55] Fabian ID, Stacey AW, Bowman R (2021). Retinoblastoma management during the COVID-19 pandemic: a report by the Global Retinoblastoma Study Group including 194 centers from 94 countries. Pediatr Blood Cancer.

[56] Hadjistilianou D, Barchitta M, Defrancesco S (2020). Covid-19 and retinoblastoma: how we manage it. Eur J Ophthalmol.

[57] Majeed A, Wright T, Guo B, Arora RS, Lam CG, Martiniuk AL (2022). The global impact of COVID-19 on childhood cancer outcomes and care delivery-a systematic review. Front Oncol.

[58] Gelkopf MJ, Chang TE, Zhang Y (2019). Parental coping with retinoblastoma diagnosis. J Psychosoc Oncol.

[59] Xiao W, Ji X, Ye H (2020). Parent knowledge of screening and genetic testing in retinoblastoma. J Ophthalmol.

[60] Soliman SE, Eldomiaty W, Goweida MB, Dowidar A (2017). Clinical presentation of retinoblastoma in Alexandria: a step toward earlier diagnosis. Saudi J Ophthalmol.

[61] Corsi DJ, Bassani DG, Kumar R (2009). Gender inequity and age-appropriate immunization coverage in India from 1992 to 2006. BMC Int Health Hum Rights.

[62] Jha P, Kumar R, Vasa P, Dhingra N, Thiruchelvam D, Moineddin R (2006). Low female [corrected]-to-male [corrected] sex ratio of children born in India: national survey of 1.1 million households. Lancet.

[63] Khera R, Jain S, Lodha R, Ramakrishnan S (2014). Gender bias in child care and child health: global patterns. Arch Dis Child.

[64] Attané I (2009). The determinants of discrimination against daughters in China: evidence from a provincial-level analysis. Popul Stud (Camb).

[65] Dancer D, Rammohan A, Smith MD (2008). Infant mortality and child nutrition in Bangladesh. Health Econ.

[66] Mitra AK, Rahman MM, Fuchs GJ (2000). Risk factors and gender differentials for death among children hospitalized with diarrhoea in Bangladesh. J Health Popul Nutr.

[67] Nuruddin R, Hadden WC, Petersen MR, Lim MK (2009). Does child gender determine household decision for health care in rural Thatta, Pakistan?. J Public Health (Oxf).

[68] Pokhrel S, Snow R, Dong H, Hidayat B, Flessa S, Sauerborn R (2005). Gender role and child health care utilization in Nepal. Health Policy.

[69] Bhargav A, Singh U, Trehan A, Zadeng Z, Bansal D (2017). Female sex, bilateral disease, age below 3 years, and apprehension for enucleation contribute to treatment abandonment in retinoblastoma. J Pediatr Hematol Oncol.

[70] The Lancet Oncology (2022). Oncologists in social media-what are the limits?. Lancet Oncol.

