# Supplementary material for: A Machine Learning Pipeline to Analyze Global Sentiment and Factors Influencing Retinoblastoma Treatment Hesitancy: Observational Infodemiology Study
Source: J Med Internet Res. 2026 Aug 11;28:e73364. doi: 10.2196/73364 (PMC13460677; doi:10.2196/73364)
Supplement: Supplementary file 1 [file jmir-v28-e73364-s001.docx]

**Supplementary Table 1: Summary of Existing Work on Social Media and Pediatric Cancer and Rare Disease**

| Literature | Author | Platforms | Summary |
| --- | --- | --- | --- |
| Exploring the use of social media and online methods to engage persons with lived experience and healthcare professionals in creating research agendas: Lessons from a pediatric cancer research priority-setting partnership [1] | Hwang et al. (2024) | Facebook, Instagram | The research team designed priority-setting partnership to use social media platform to develop pediatric cancer research agenda. They developed framework for preparation, target audience identification, campaign content design, campaign operation, and evaluation, and provided recommendation for future social media campaigns. Cost analysis has also been performed. By interacting with social media users locally and worldwide, a community-specified research agenda has been formulated. |
| Insights From Diverse Perspectives on Social Media Messages to Inform Young Adults With Cancer About Clinical Trials: Focus Group Study [2] | Beauchemin et al. (2025) | Facebook support groups, Instagram | The study initiated 5 focus groups in 2023 for young adult patients across United States.  The study identified social media as important channel for informal outreach, and identified key thematic barriers including lack of concise and readily available information, cost assumptions and coverage of insurance, financial burden, e.t.c. |
| Harnessing Social Media to Develop Conceptual Domains of Quality of Life for Adolescents With Advanced Cancer [3] | Johnson et al. (2025) | TikTok, Facebook, Instagram, YouTube, Blogger, Blogspot, and Reddit. | The study recorded 235 social media from 14 participants. Posts were used to provide information on Quality of life for teenagers with advanced cancer. The study identified 6 key domains of quality of life concerns and impact, namely “perceived health”, ”the lived body”, “emotional wellbeing”, “normalcy”, “purpose and direction” and “reorientation” |
| Adolescent childhood cancer survivors talking about cancer: A socioecological perspective [4] | Honda et al. (2025) | Offline surveys | Participants were recruited from pediatric cancer outpatient clinic. Interviews were performed to summarise usage pattern of social media by adolescent cancer survivors. The study reported that proactive sharing of cancer-related information was limited, and discussions focused mainly on emotional support. Communication pattern was influenced by parents, and was also influenced by factors from different levels of socialization. |
| #MadelungDeformity: Insights Into a Rare Congenital Difference Using Social Media [5] | Peymmani et al (2023) | Online surveys on Facebook and Instagram | Survey was created with reference to Patient-Reported Outcomes Measurement Information System (PROMIS) short forms and distributed on Facebook and Instagram for understanding the clinical spectrum and presentation of a rare condition. The study identified substantial impact on all aspects of health, including physical, mental and social, after surgical treatment. The authors concluded that the rarity of condition can be overcome by leveraging global reach of major social media sites. |
| Social Media and Website Use: The Experiences of Parents and Carers Accessing Care at the Oxford Craniofacial Unit [6] | Kilcoyne et al (2023) | Offline surveys | 83 parents were included at multidisciplinary craniofacial clinics. Usage pattern of social media and website was investigated using questionnaire by Khouri and colleagues (2016) and Huggons and colleagues (2019). 93% and 72% of parents used internet and social media for information seeking on the craniofacial conditions. Facebook was reported as the most helpful and popular source of information among all social networking sites. Most parents started using social media for information after diagnosis was made, followed by before craniofacial surgery. The study highlighted the important role of social media for information dissemination. |
| Comorbidities and emotions - unpacking the sentiments of pediatric patients with multiple long-term conditions through social media feedback: A large language model-driven study [7] | Oluwalade et al (2025) | Care Opinion platform | 389 narratives were extracted from the Care Opinion platform. Texts were analyzed by CoEmoBERT, a distilroberta-base transformer model. Most prevalent sentiment was sad and fear, with 93.8% of negative sentiment. Negative sentiment found to be related to severe comorbidities. Exacerbation of negative sentiment by COVID-19 pandemic was noted. |
| Pediatric Cancer Communication on Twitter: Natural Language Processing and Qualitative Content Analysis [8] | Lau et al (2024) | Twitter | 161,135 pediatric cancer related tweets were extracted. Sentiment analysis was performed using the Bing, AFINN, and NRC lexicons. A net positive sentiment was identified. Qualitative analysis with manual coding was performed to identify popular topics. These included the use of Twitter to promote awareness of pediatric cancer and sharing of personal experiences, as well as promotion of research findings and policies. |

1.	Hwang, K., et al., *Exploring the use of social media and online methods to engage persons with lived experience and healthcare professionals in creating research agendas: Lessons from a pediatric cancer research priority-setting partnership.* PLOS Digit Health, 2024. **3**(1): p. e0000181.

2.	Beauchemin, M.P., et al., *Insights From Diverse Perspectives on Social Media Messages to Inform Young Adults With Cancer About Clinical Trials: Focus Group Study.* JMIR Form Res, 2025. **9**: p. e64265.

3.	Johnson, A. and K. Widger, *Harnessing Social Media to Develop Conceptual Domains of Quality of Life for Adolescents With Advanced Cancer.* Psychooncology, 2025. **34**(5): p. e70155.

4.	Honda, N., et al., *Adolescent childhood cancer survivors talking about cancer: A socioecological perspective.* Asia-Pacific Journal of Oncology Nursing, 2025. **12**: p. 100676.

5.	Peymani, A., et al., *#MadelungDeformity: Insights Into a Rare Congenital Difference Using Social Media.* Hand (N Y), 2023. **18**(2_suppl): p. 24s-31s.

6.	Kilcoyne, S., et al., *Social Media and Website Use: The Experiences of Parents and Carers Accessing Care at the Oxford Craniofacial Unit.* Journal of Craniofacial Surgery, 2024. **35**(1).

7.	Oluwalade, T.I., et al., *Comorbidities and emotions - unpacking the sentiments of pediatric patients with multiple long-term conditions through social media feedback: A large language model-driven study.* J Affect Disord, 2025. **388**: p. 119752.

8.	Lau, N., et al., *Pediatric Cancer Communication on Twitter: Natural Language Processing and Qualitative Content Analysis.* JMIR Cancer, 2024. **10**: p. e52061.

**Supplementary Table 2: Overall Cross-validation Statistics for BERT Model for Text Relevance Classification**

**Supplementary Table 2a: Summary of Cross-validation Statistics for Text Relevance Classification**

| Learning Rate | Batch Size | Epochs | AUC | F1 Score | Accuracy |
| --- | --- | --- | --- | --- | --- |
| 2E-05 | 16 | 1 | 0.978 [0.969-0.987] | 0.940 [0.922-0.958] | 94.0% [92.2%-95.8%] |
| 2E-05 | 16 | 2 | 0.985 [0.976-0.993] | 0.948 [0.936-0.959] | 94.8% [93.7%-95.9%] |
| 2E-05 | 16 | 3 | 0.985 [0.977-0.993] | 0.952 [0.935-0.969] | 95.2% [93.6%-96.9%] |
| 2E-05 | 16 | 4 | 0.987 [0.981-0.993] | 0.950 [0.930-0.969] | 95.0% [93.1%-96.9%] |
| 2E-05 | 16 | 5 | 0.986 [0.979-0.992] | 0.943 [0.928-0.958] | 94.3% [92.9%-95.8%] |
| 2E-05 | 16 | 6 | 0.987 [0.982-0.992] | 0.944 [0.926-0.963] | 94.4% [92.6%-96.3%] |
| 2E-05 | 16 | 7 | 0.986 [0.979-0.994] | 0.944 [0.925-0.963] | 94.4% [92.6%-96.3%] |
| 2E-05 | 16 | 8 | 0.985 [0.978-0.992] | 0.957 [0.946-0.968] | 95.7% [94.6%-96.8%] |
| 2E-05 | 32 | 1 | 0.964 [0.952-0.976] | 0.903 [0.868-0.938] | 90.3% [86.9%-93.8%] |
| 2E-05 | 32 | 2 | 0.977 [0.966-0.989] | 0.943 [0.924-0.963] | 94.3% [92.4%-96.3%] |
| 2E-05 | 32 | 3 | 0.983 [0.976-0.990] | 0.949 [0.934-0.964] | 94.9% [93.4%-96.4%] |
| 2E-05 | 32 | 4 | 0.988 [0.983-0.993] | 0.951 [0.931-0.971] | 95.1% [93.1%-97.1%] |
| 2E-05 | 32 | 5 | 0.984 [0.977-0.992] | 0.947 [0.927-0.966] | 94.7% [92.8%-96.6%] |
| 2E-05 | 32 | 6 | 0.982 [0.973-0.992] | 0.951 [0.936-0.966] | 95.1% [93.7%-96.6%] |
| 2E-05 | 32 | 7 | 0.986 [0.979-0.993] | 0.942 [0.924-0.960] | 94.2% [92.5%-95.9%] |
| 2E-05 | 32 | 8 | 0.985 [0.979-0.991] | 0.951 [0.930-0.972] | 95.1% [93.1%-97.1%] |
| 5E-05 | 16 | 1 | 0.976 [0.965-0.988] | 0.926 [0.881-0.972] | 92.8% [88.6%-97.0%] |
| 5E-05 | 16 | 2 | 0.980 [0.969-0.991] | 0.942 [0.922-0.962] | 94.2% [92.2%-96.2%] |
| 5E-05 | 16 | 3 | 0.983 [0.974-0.991] | 0.961 [0.949-0.973] | 96.1% [94.9%-97.3%] |
| 5E-05 | 16 | 4 | 0.986 [0.977-0.996] | 0.952 [0.932-0.972] | 95.2% [93.2%-97.2%] |
| 5E-05 | 16 | 5 | 0.981 [0.972-0.991] | 0.954 [0.941-0.968] | 95.4% [94.1%-96.8%] |
| 5E-05 | 16 | 6 | 0.983 [0.977-0.990] | 0.954 [0.936-0.973] | 95.4% [93.6%-97.3%] |
| 5E-05 | 16 | 7 | 0.979 [0.969-0.989] | 0.956 [0.940-0.971] | 95.6% [94.1%-97.1%] |
| 5E-05 | 16 | 8 | 0.982 [0.975-0.989] | 0.953 [0.941-0.966] | 95.3% [94.1%-96.6%] |
| 5E-05 | 32 | 1 | 0.976 [0.963-0.989] | 0.937 [0.913-0.962] | 93.8% [91.4%-96.2%] |
| 5E-05 | 32 | 2 | 0.986 [0.978-0.994] | 0.949 [0.935-0.962] | 94.9% [93.5%-96.3%] |
| 5E-05 | 32 | 3 | 0.985 [0.977-0.994] | 0.954 [0.941-0.968] | 95.4% [94.1%-96.8%] |
| 5E-05 | 32 | 4 | 0.984 [0.975-0.994] | 0.959 [0.943-0.974] | 95.9% [94.3%-97.4%] |
| 5E-05 | 32 | 5 | 0.987 [0.983-0.991] | 0.957 [0.944-0.969] | 95.7% [94.5%-96.9%] |
| 5E-05 | 32 | 6 | 0.984 [0.977-0.991] | 0.952 [0.942-0.963] | 95.2% [94.2%-96.3%] |
| 5E-05 | 32 | 7 | 0.983 [0.974-0.992] | 0.953 [0.933-0.973] | 95.3% [93.4%-97.3%] |
| 5E-05 | 32 | 8 | 0.978 [0.968-0.988] | 0.951 [0.935-0.967] | 95.1% [93.6%-96.7%] |

**Supplementary Table 2b: Per-fold Performance of Cross-validation Statistics for Text Relevance Classification**

| Fold | Learning Rate | Batch Size | Epochs | AUC | F1 Score | Accuracy |
| --- | --- | --- | --- | --- | --- | --- |
| 1 | 2.00E-05 | 16 | 1 | 0.9628 | 0.9333 | 0.9333 |
| 1 | 2.00E-05 | 16 | 2 | 0.9915 | 0.9668 | 0.9667 |
| 1 | 2.00E-05 | 16 | 3 | 1.0000 | 0.9889 | 0.9889 |
| 1 | 2.00E-05 | 16 | 4 | 0.9980 | 0.9665 | 0.9667 |
| 1 | 2.00E-05 | 16 | 5 | 1.0000 | 0.9778 | 0.9778 |
| 1 | 2.00E-05 | 16 | 6 | 1.0000 | 0.9778 | 0.9778 |
| 1 | 2.00E-05 | 16 | 7 | 0.9980 | 0.9778 | 0.9778 |
| 1 | 2.00E-05 | 16 | 8 | 1.0000 | 0.9778 | 0.9778 |
| 1 | 2.00E-05 | 32 | 1 | 0.9502 | 0.8628 | 0.8667 |
| 1 | 2.00E-05 | 32 | 2 | 0.9965 | 0.9557 | 0.9556 |
| 1 | 2.00E-05 | 32 | 3 | 1.0000 | 0.9668 | 0.9667 |
| 1 | 2.00E-05 | 32 | 4 | 1.0000 | 0.9889 | 0.9889 |
| 1 | 2.00E-05 | 32 | 5 | 1.0000 | 1.0000 | 1.0000 |
| 1 | 2.00E-05 | 32 | 6 | 0.9980 | 0.9668 | 0.9667 |
| 1 | 2.00E-05 | 32 | 7 | 0.9990 | 0.9668 | 0.9667 |
| 1 | 2.00E-05 | 32 | 8 | 0.9985 | 0.9889 | 0.9889 |
| 1 | 5.00E-05 | 16 | 1 | 0.9970 | 0.9226 | 0.9222 |
| 1 | 5.00E-05 | 16 | 2 | 1.0000 | 0.9889 | 0.9889 |
| 1 | 5.00E-05 | 16 | 3 | 0.9940 | 0.9557 | 0.9556 |
| 1 | 5.00E-05 | 16 | 4 | 1.0000 | 1.0000 | 1.0000 |
| 1 | 5.00E-05 | 16 | 5 | 0.9970 | 0.9668 | 0.9667 |
| 1 | 5.00E-05 | 16 | 6 | 1.0000 | 1.0000 | 1.0000 |
| 1 | 5.00E-05 | 16 | 7 | 0.9980 | 0.9778 | 0.9778 |
| 1 | 5.00E-05 | 16 | 8 | 0.9955 | 0.9668 | 0.9667 |
| 1 | 5.00E-05 | 32 | 1 | 0.9970 | 0.9438 | 0.9444 |
| 1 | 5.00E-05 | 32 | 2 | 0.9945 | 0.9668 | 0.9667 |
| 1 | 5.00E-05 | 32 | 3 | 0.9945 | 0.9668 | 0.9667 |
| 1 | 5.00E-05 | 32 | 4 | 0.9990 | 0.9777 | 0.9778 |
| 1 | 5.00E-05 | 32 | 5 | 0.9965 | 0.9668 | 0.9667 |
| 1 | 5.00E-05 | 32 | 6 | 0.9925 | 0.9668 | 0.9667 |
| 1 | 5.00E-05 | 32 | 7 | 0.9980 | 0.9778 | 0.9778 |
| 1 | 5.00E-05 | 32 | 8 | 0.9970 | 0.9778 | 0.9778 |
| 2 | 2.00E-05 | 16 | 1 | 0.9955 | 0.9446 | 0.9444 |
| 2 | 2.00E-05 | 16 | 2 | 0.9935 | 0.9336 | 0.9333 |
| 2 | 2.00E-05 | 16 | 3 | 0.9804 | 0.9441 | 0.9444 |
| 2 | 2.00E-05 | 16 | 4 | 0.9904 | 0.9226 | 0.9222 |
| 2 | 2.00E-05 | 16 | 5 | 0.9864 | 0.9113 | 0.9111 |
| 2 | 2.00E-05 | 16 | 6 | 0.9829 | 0.9226 | 0.9222 |
| 2 | 2.00E-05 | 16 | 7 | 0.9940 | 0.9557 | 0.9556 |
| 2 | 2.00E-05 | 16 | 8 | 0.9930 | 0.9446 | 0.9444 |
| 2 | 2.00E-05 | 32 | 1 | 0.9397 | 0.8009 | 0.8000 |
| 2 | 2.00E-05 | 32 | 2 | 0.9739 | 0.9115 | 0.9111 |
| 2 | 2.00E-05 | 32 | 3 | 0.9794 | 0.9111 | 0.9111 |
| 2 | 2.00E-05 | 32 | 4 | 0.9864 | 0.9225 | 0.9222 |
| 2 | 2.00E-05 | 32 | 5 | 0.9899 | 0.9226 | 0.9222 |
| 2 | 2.00E-05 | 32 | 6 | 0.9729 | 0.9226 | 0.9222 |
| 2 | 2.00E-05 | 32 | 7 | 0.9904 | 0.9336 | 0.9333 |
| 2 | 2.00E-05 | 32 | 8 | 0.9920 | 0.9335 | 0.9333 |
| 2 | 5.00E-05 | 16 | 1 | 0.9879 | 0.9003 | 0.9000 |
| 2 | 5.00E-05 | 16 | 2 | 0.9899 | 0.9115 | 0.9111 |
| 2 | 5.00E-05 | 16 | 3 | 0.9965 | 0.9889 | 0.9889 |
| 2 | 5.00E-05 | 16 | 4 | 0.9910 | 0.9225 | 0.9222 |
| 2 | 5.00E-05 | 16 | 5 | 0.9779 | 0.9446 | 0.9444 |
| 2 | 5.00E-05 | 16 | 6 | 0.9693 | 0.9556 | 0.9556 |
| 2 | 5.00E-05 | 16 | 7 | 0.9668 | 0.9446 | 0.9444 |
| 2 | 5.00E-05 | 16 | 8 | 0.9759 | 0.9446 | 0.9444 |
| 2 | 5.00E-05 | 32 | 1 | 0.9804 | 0.9226 | 0.9222 |
| 2 | 5.00E-05 | 32 | 2 | 0.9884 | 0.9221 | 0.9222 |
| 2 | 5.00E-05 | 32 | 3 | 0.9975 | 0.9336 | 0.9333 |
| 2 | 5.00E-05 | 32 | 4 | 0.9849 | 0.9336 | 0.9333 |
| 2 | 5.00E-05 | 32 | 5 | 0.9945 | 0.9778 | 0.9778 |
| 2 | 5.00E-05 | 32 | 6 | 0.9799 | 0.9333 | 0.9333 |
| 2 | 5.00E-05 | 32 | 7 | 0.9975 | 0.9778 | 0.9778 |
| 2 | 5.00E-05 | 32 | 8 | 0.9884 | 0.9225 | 0.9222 |
| 3 | 2.00E-05 | 16 | 1 | 0.9741 | 0.9108 | 0.9111 |
| 3 | 2.00E-05 | 16 | 2 | 0.9897 | 0.9554 | 0.9556 |
| 3 | 2.00E-05 | 16 | 3 | 0.9771 | 0.9554 | 0.9556 |
| 3 | 2.00E-05 | 16 | 4 | 0.9847 | 0.8983 | 0.9000 |
| 3 | 2.00E-05 | 16 | 5 | 0.9832 | 0.9214 | 0.9222 |
| 3 | 2.00E-05 | 16 | 6 | 0.9837 | 0.9214 | 0.9222 |
| 3 | 2.00E-05 | 16 | 7 | 0.9796 | 0.9556 | 0.9556 |
| 3 | 2.00E-05 | 16 | 8 | 0.9676 | 0.9444 | 0.9444 |
| 3 | 2.00E-05 | 32 | 1 | 0.9706 | 0.9225 | 0.9222 |
| 3 | 2.00E-05 | 32 | 2 | 0.9796 | 0.9223 | 0.9222 |
| 3 | 2.00E-05 | 32 | 3 | 0.9681 | 0.9446 | 0.9444 |
| 3 | 2.00E-05 | 32 | 4 | 0.9791 | 0.9336 | 0.9333 |
| 3 | 2.00E-05 | 32 | 5 | 0.9746 | 0.9445 | 0.9444 |
| 3 | 2.00E-05 | 32 | 6 | 0.9842 | 0.9445 | 0.9444 |
| 3 | 2.00E-05 | 32 | 7 | 0.9761 | 0.9335 | 0.9333 |
| 3 | 2.00E-05 | 32 | 8 | 0.9776 | 0.9445 | 0.9444 |
| 3 | 5.00E-05 | 16 | 1 | 0.9706 | 0.9446 | 0.9444 |
| 3 | 5.00E-05 | 16 | 2 | 0.9605 | 0.9557 | 0.9556 |
| 3 | 5.00E-05 | 16 | 3 | 0.9736 | 0.9554 | 0.9556 |
| 3 | 5.00E-05 | 16 | 4 | 0.9801 | 0.9444 | 0.9444 |
| 3 | 5.00E-05 | 16 | 5 | 0.9615 | 0.9554 | 0.9556 |
| 3 | 5.00E-05 | 16 | 6 | 0.9781 | 0.9556 | 0.9556 |
| 3 | 5.00E-05 | 16 | 7 | 0.9761 | 0.9557 | 0.9556 |
| 3 | 5.00E-05 | 16 | 8 | 0.9696 | 0.9328 | 0.9333 |
| 3 | 5.00E-05 | 32 | 1 | 0.9776 | 0.8628 | 0.8667 |
| 3 | 5.00E-05 | 32 | 2 | 0.9832 | 0.9557 | 0.9556 |
| 3 | 5.00E-05 | 32 | 3 | 0.9867 | 0.9441 | 0.9444 |
| 3 | 5.00E-05 | 32 | 4 | 0.9781 | 0.9441 | 0.9444 |
| 3 | 5.00E-05 | 32 | 5 | 0.9837 | 0.9445 | 0.9444 |
| 3 | 5.00E-05 | 32 | 6 | 0.9756 | 0.9446 | 0.9444 |
| 3 | 5.00E-05 | 32 | 7 | 0.9696 | 0.9444 | 0.9444 |
| 3 | 5.00E-05 | 32 | 8 | 0.9786 | 0.9554 | 0.9556 |
| 4 | 2.00E-05 | 16 | 1 | 0.9849 | 0.9104 | 0.9111 |
| 4 | 2.00E-05 | 16 | 2 | 0.9950 | 0.9438 | 0.9444 |
| 4 | 2.00E-05 | 16 | 3 | 0.9960 | 0.9552 | 0.9556 |
| 4 | 2.00E-05 | 16 | 4 | 0.9940 | 0.9667 | 0.9667 |
| 4 | 2.00E-05 | 16 | 5 | 0.9869 | 0.9328 | 0.9333 |
| 4 | 2.00E-05 | 16 | 6 | 0.9904 | 0.9441 | 0.9444 |
| 4 | 2.00E-05 | 16 | 7 | 0.9910 | 0.9093 | 0.9111 |
| 4 | 2.00E-05 | 16 | 8 | 0.9920 | 0.9667 | 0.9667 |
| 4 | 2.00E-05 | 32 | 1 | 0.9668 | 0.8507 | 0.8556 |
| 4 | 2.00E-05 | 32 | 2 | 0.9789 | 0.9218 | 0.9222 |
| 4 | 2.00E-05 | 32 | 3 | 0.9945 | 0.9328 | 0.9333 |
| 4 | 2.00E-05 | 32 | 4 | 0.9945 | 0.9093 | 0.9111 |
| 4 | 2.00E-05 | 32 | 5 | 0.9920 | 0.9666 | 0.9667 |
| 4 | 2.00E-05 | 32 | 6 | 0.9955 | 0.9324 | 0.9333 |
| 4 | 2.00E-05 | 32 | 7 | 0.9904 | 0.9331 | 0.9333 |
| 4 | 2.00E-05 | 32 | 8 | 0.9864 | 0.9666 | 0.9667 |
| 4 | 5.00E-05 | 16 | 1 | 0.9578 | 0.7611 | 0.7778 |
| 4 | 5.00E-05 | 16 | 2 | 0.9894 | 0.9666 | 0.9667 |
| 4 | 5.00E-05 | 16 | 3 | 0.9718 | 0.9556 | 0.9556 |
| 4 | 5.00E-05 | 16 | 4 | 0.9940 | 0.9777 | 0.9778 |
| 4 | 5.00E-05 | 16 | 5 | 0.9925 | 0.9554 | 0.9556 |
| 4 | 5.00E-05 | 16 | 6 | 0.9829 | 0.9104 | 0.9111 |
| 4 | 5.00E-05 | 16 | 7 | 0.9824 | 0.9104 | 0.9111 |
| 4 | 5.00E-05 | 16 | 8 | 0.9759 | 0.9554 | 0.9556 |
| 4 | 5.00E-05 | 32 | 1 | 0.9844 | 0.9667 | 0.9667 |
| 4 | 5.00E-05 | 32 | 2 | 0.9930 | 0.9444 | 0.9444 |
| 4 | 5.00E-05 | 32 | 3 | 0.9779 | 0.9556 | 0.9556 |
| 4 | 5.00E-05 | 32 | 4 | 0.9769 | 0.9328 | 0.9333 |
| 4 | 5.00E-05 | 32 | 5 | 0.9824 | 0.9666 | 0.9667 |
| 4 | 5.00E-05 | 32 | 6 | 0.9904 | 0.9331 | 0.9333 |
| 4 | 5.00E-05 | 32 | 7 | 0.9658 | 0.9556 | 0.9556 |
| 4 | 5.00E-05 | 32 | 8 | 0.9739 | 0.9218 | 0.9222 |
| 5 | 2.00E-05 | 16 | 1 | 0.9725 | 0.9333 | 0.9333 |
| 5 | 2.00E-05 | 16 | 2 | 0.9720 | 0.9556 | 0.9556 |
| 5 | 2.00E-05 | 16 | 3 | 0.9785 | 0.9442 | 0.9444 |
| 5 | 2.00E-05 | 16 | 4 | 0.9780 | 0.9442 | 0.9444 |
| 5 | 2.00E-05 | 16 | 5 | 0.9920 | 0.9444 | 0.9444 |
| 5 | 2.00E-05 | 16 | 6 | 0.9825 | 0.9218 | 0.9222 |
| 5 | 2.00E-05 | 16 | 7 | 0.9730 | 0.9444 | 0.9444 |
| 5 | 2.00E-05 | 16 | 8 | 0.9775 | 0.9442 | 0.9444 |
| 5 | 2.00E-05 | 32 | 1 | 0.9615 | 0.9333 | 0.9333 |
| 5 | 2.00E-05 | 32 | 2 | 0.9490 | 0.9444 | 0.9444 |
| 5 | 2.00E-05 | 32 | 3 | 0.9805 | 0.9444 | 0.9444 |
| 5 | 2.00E-05 | 32 | 4 | 0.9855 | 0.9331 | 0.9333 |
| 5 | 2.00E-05 | 32 | 5 | 0.9715 | 0.9329 | 0.9333 |
| 5 | 2.00E-05 | 32 | 6 | 0.9705 | 0.9444 | 0.9444 |
| 5 | 2.00E-05 | 32 | 7 | 0.9915 | 0.9331 | 0.9333 |
| 5 | 2.00E-05 | 32 | 8 | 0.9805 | 0.9556 | 0.9556 |
| 5 | 5.00E-05 | 16 | 1 | 0.9550 | 0.9333 | 0.9333 |
| 5 | 5.00E-05 | 16 | 2 | 0.9725 | 0.9223 | 0.9222 |
| 5 | 5.00E-05 | 16 | 3 | 0.9605 | 0.9554 | 0.9556 |
| 5 | 5.00E-05 | 16 | 4 | 0.9855 | 0.9554 | 0.9556 |
| 5 | 5.00E-05 | 16 | 5 | 0.9665 | 0.9442 | 0.9444 |
| 5 | 5.00E-05 | 16 | 6 | 0.9835 | 0.9444 | 0.9444 |
| 5 | 5.00E-05 | 16 | 7 | 0.9760 | 0.9554 | 0.9556 |
| 5 | 5.00E-05 | 16 | 8 | 0.9920 | 0.9442 | 0.9444 |
| 5 | 5.00E-05 | 32 | 1 | 0.9810 | 0.9444 | 0.9444 |
| 5 | 5.00E-05 | 32 | 2 | 0.9600 | 0.9444 | 0.9444 |
| 5 | 5.00E-05 | 32 | 3 | 0.9845 | 0.9552 | 0.9556 |
| 5 | 5.00E-05 | 32 | 4 | 0.9735 | 0.9556 | 0.9556 |
| 5 | 5.00E-05 | 32 | 5 | 0.9815 | 0.9444 | 0.9444 |
| 5 | 5.00E-05 | 32 | 6 | 0.9755 | 0.9556 | 0.9556 |
| 5 | 5.00E-05 | 32 | 7 | 0.9820 | 0.9331 | 0.9333 |
| 5 | 5.00E-05 | 32 | 8 | 0.9850 | 0.9444 | 0.9444 |
| 6 | 2.00E-05 | 16 | 1 | 0.9840 | 0.9778 | 0.9778 |
| 6 | 2.00E-05 | 16 | 2 | 0.9875 | 0.9778 | 0.9778 |
| 6 | 2.00E-05 | 16 | 3 | 0.9945 | 0.9666 | 0.9667 |
| 6 | 2.00E-05 | 16 | 4 | 0.9965 | 0.9778 | 0.9778 |
| 6 | 2.00E-05 | 16 | 5 | 0.9905 | 0.9666 | 0.9667 |
| 6 | 2.00E-05 | 16 | 6 | 0.9865 | 0.9666 | 0.9667 |
| 6 | 2.00E-05 | 16 | 7 | 0.9965 | 0.9666 | 0.9667 |
| 6 | 2.00E-05 | 16 | 8 | 0.9935 | 0.9666 | 0.9667 |
| 6 | 2.00E-05 | 32 | 1 | 0.9790 | 0.9331 | 0.9333 |
| 6 | 2.00E-05 | 32 | 2 | 0.9890 | 0.9889 | 0.9889 |
| 6 | 2.00E-05 | 32 | 3 | 0.9880 | 0.9778 | 0.9778 |
| 6 | 2.00E-05 | 32 | 4 | 0.9925 | 0.9889 | 0.9889 |
| 6 | 2.00E-05 | 32 | 5 | 0.9900 | 0.9100 | 0.9111 |
| 6 | 2.00E-05 | 32 | 6 | 0.9920 | 0.9889 | 0.9889 |
| 6 | 2.00E-05 | 32 | 7 | 0.9815 | 0.9666 | 0.9667 |
| 6 | 2.00E-05 | 32 | 8 | 0.9890 | 0.9666 | 0.9667 |
| 6 | 5.00E-05 | 16 | 1 | 0.9900 | 0.9778 | 0.9778 |
| 6 | 5.00E-05 | 16 | 2 | 0.9915 | 0.9100 | 0.9111 |
| 6 | 5.00E-05 | 16 | 3 | 0.9945 | 0.9778 | 0.9778 |
| 6 | 5.00E-05 | 16 | 4 | 0.9935 | 0.9666 | 0.9667 |
| 6 | 5.00E-05 | 16 | 5 | 0.9915 | 0.9778 | 0.9778 |
| 6 | 5.00E-05 | 16 | 6 | 0.9930 | 0.9666 | 0.9667 |
| 6 | 5.00E-05 | 16 | 7 | 0.9850 | 0.9889 | 0.9889 |
| 6 | 5.00E-05 | 16 | 8 | 0.9955 | 0.9666 | 0.9667 |
| 6 | 5.00E-05 | 32 | 1 | 0.9715 | 0.9778 | 0.9778 |
| 6 | 5.00E-05 | 32 | 2 | 0.9940 | 0.9889 | 0.9889 |
| 6 | 5.00E-05 | 32 | 3 | 0.9960 | 0.9889 | 0.9889 |
| 6 | 5.00E-05 | 32 | 4 | 0.9950 | 0.9666 | 0.9667 |
| 6 | 5.00E-05 | 32 | 5 | 0.9915 | 0.9666 | 0.9667 |
| 6 | 5.00E-05 | 32 | 6 | 0.9905 | 0.9442 | 0.9444 |
| 6 | 5.00E-05 | 32 | 7 | 0.9905 | 0.9666 | 0.9667 |
| 6 | 5.00E-05 | 32 | 8 | 0.9820 | 0.9778 | 0.9778 |
| 7 | 2.00E-05 | 16 | 1 | 0.9635 | 0.9113 | 0.9111 |
| 7 | 2.00E-05 | 16 | 2 | 0.9600 | 0.9335 | 0.9333 |
| 7 | 2.00E-05 | 16 | 3 | 0.9675 | 0.9446 | 0.9444 |
| 7 | 2.00E-05 | 16 | 4 | 0.9735 | 0.9223 | 0.9222 |
| 7 | 2.00E-05 | 16 | 5 | 0.9655 | 0.9335 | 0.9333 |
| 7 | 2.00E-05 | 16 | 6 | 0.9725 | 0.9111 | 0.9111 |
| 7 | 2.00E-05 | 16 | 7 | 0.9675 | 0.9224 | 0.9222 |
| 7 | 2.00E-05 | 16 | 8 | 0.9745 | 0.9335 | 0.9333 |
| 7 | 2.00E-05 | 32 | 1 | 0.9510 | 0.9111 | 0.9111 |
| 7 | 2.00E-05 | 32 | 2 | 0.9570 | 0.9225 | 0.9222 |
| 7 | 2.00E-05 | 32 | 3 | 0.9775 | 0.9335 | 0.9333 |
| 7 | 2.00E-05 | 32 | 4 | 0.9785 | 0.9335 | 0.9333 |
| 7 | 2.00E-05 | 32 | 5 | 0.9665 | 0.9225 | 0.9222 |
| 7 | 2.00E-05 | 32 | 6 | 0.9545 | 0.9446 | 0.9444 |
| 7 | 2.00E-05 | 32 | 7 | 0.9665 | 0.9445 | 0.9444 |
| 7 | 2.00E-05 | 32 | 8 | 0.9700 | 0.9335 | 0.9333 |
| 7 | 5.00E-05 | 16 | 1 | 0.9660 | 0.9225 | 0.9222 |
| 7 | 5.00E-05 | 16 | 2 | 0.9595 | 0.9223 | 0.9222 |
| 7 | 5.00E-05 | 16 | 3 | 0.9810 | 0.9335 | 0.9333 |
| 7 | 5.00E-05 | 16 | 4 | 0.9550 | 0.9223 | 0.9222 |
| 7 | 5.00E-05 | 16 | 5 | 0.9605 | 0.9224 | 0.9222 |
| 7 | 5.00E-05 | 16 | 6 | 0.9710 | 0.9224 | 0.9222 |
| 7 | 5.00E-05 | 16 | 7 | 0.9470 | 0.9446 | 0.9444 |
| 7 | 5.00E-05 | 16 | 8 | 0.9725 | 0.9335 | 0.9333 |
| 7 | 5.00E-05 | 32 | 1 | 0.9560 | 0.9225 | 0.9222 |
| 7 | 5.00E-05 | 32 | 2 | 0.9775 | 0.9335 | 0.9333 |
| 7 | 5.00E-05 | 32 | 3 | 0.9580 | 0.9335 | 0.9333 |
| 7 | 5.00E-05 | 32 | 4 | 0.9570 | 0.9335 | 0.9333 |
| 7 | 5.00E-05 | 32 | 5 | 0.9840 | 0.9446 | 0.9444 |
| 7 | 5.00E-05 | 32 | 6 | 0.9630 | 0.9445 | 0.9444 |
| 7 | 5.00E-05 | 32 | 7 | 0.9690 | 0.9108 | 0.9111 |
| 7 | 5.00E-05 | 32 | 8 | 0.9585 | 0.9446 | 0.9444 |
| 8 | 2.00E-05 | 16 | 1 | 0.9695 | 0.9446 | 0.9444 |
| 8 | 2.00E-05 | 16 | 2 | 0.9855 | 0.9442 | 0.9444 |
| 8 | 2.00E-05 | 16 | 3 | 0.9860 | 0.9554 | 0.9556 |
| 8 | 2.00E-05 | 16 | 4 | 0.9835 | 0.9667 | 0.9667 |
| 8 | 2.00E-05 | 16 | 5 | 0.9805 | 0.9556 | 0.9556 |
| 8 | 2.00E-05 | 16 | 6 | 0.9910 | 0.9778 | 0.9778 |
| 8 | 2.00E-05 | 16 | 7 | 0.9855 | 0.9442 | 0.9444 |
| 8 | 2.00E-05 | 16 | 8 | 0.9885 | 0.9778 | 0.9778 |
| 8 | 2.00E-05 | 32 | 1 | 0.9510 | 0.9113 | 0.9111 |
| 8 | 2.00E-05 | 32 | 2 | 0.9660 | 0.9223 | 0.9222 |
| 8 | 2.00E-05 | 32 | 3 | 0.9825 | 0.9778 | 0.9778 |
| 8 | 2.00E-05 | 32 | 4 | 0.9860 | 0.9666 | 0.9667 |
| 8 | 2.00E-05 | 32 | 5 | 0.9825 | 0.9444 | 0.9444 |
| 8 | 2.00E-05 | 32 | 6 | 0.9825 | 0.9444 | 0.9444 |
| 8 | 2.00E-05 | 32 | 7 | 0.9825 | 0.9554 | 0.9556 |
| 8 | 2.00E-05 | 32 | 8 | 0.9835 | 0.9554 | 0.9556 |
| 8 | 5.00E-05 | 16 | 1 | 0.9805 | 0.9557 | 0.9556 |
| 8 | 5.00E-05 | 16 | 2 | 0.9825 | 0.9557 | 0.9556 |
| 8 | 5.00E-05 | 16 | 3 | 0.9815 | 0.9444 | 0.9444 |
| 8 | 5.00E-05 | 16 | 4 | 0.9925 | 0.9667 | 0.9667 |
| 8 | 5.00E-05 | 16 | 5 | 0.9870 | 0.9666 | 0.9667 |
| 8 | 5.00E-05 | 16 | 6 | 0.9870 | 0.9556 | 0.9556 |
| 8 | 5.00E-05 | 16 | 7 | 0.9890 | 0.9556 | 0.9556 |
| 8 | 5.00E-05 | 16 | 8 | 0.9785 | 0.9554 | 0.9556 |
| 8 | 5.00E-05 | 32 | 1 | 0.9345 | 0.9557 | 0.9556 |
| 8 | 5.00E-05 | 32 | 2 | 0.9880 | 0.9556 | 0.9556 |
| 8 | 5.00E-05 | 32 | 3 | 0.9800 | 0.9444 | 0.9444 |
| 8 | 5.00E-05 | 32 | 4 | 0.9935 | 0.9778 | 0.9778 |
| 8 | 5.00E-05 | 32 | 5 | 0.9885 | 0.9215 | 0.9222 |
| 8 | 5.00E-05 | 32 | 6 | 0.9935 | 0.9778 | 0.9778 |
| 8 | 5.00E-05 | 32 | 7 | 0.9950 | 0.9778 | 0.9778 |
| 8 | 5.00E-05 | 32 | 8 | 0.9515 | 0.9329 | 0.9333 |
| 9 | 2.00E-05 | 16 | 1 | 0.9735 | 0.9667 | 0.9667 |
| 9 | 2.00E-05 | 16 | 2 | 0.9765 | 0.9325 | 0.9333 |
| 9 | 2.00E-05 | 16 | 3 | 0.9750 | 0.8978 | 0.9000 |
| 9 | 2.00E-05 | 16 | 4 | 0.9795 | 0.9778 | 0.9778 |
| 9 | 2.00E-05 | 16 | 5 | 0.9845 | 0.9329 | 0.9333 |
| 9 | 2.00E-05 | 16 | 6 | 0.9900 | 0.9325 | 0.9333 |
| 9 | 2.00E-05 | 16 | 7 | 0.9860 | 0.8978 | 0.9000 |
| 9 | 2.00E-05 | 16 | 8 | 0.9850 | 0.9554 | 0.9556 |
| 9 | 2.00E-05 | 32 | 1 | 0.9780 | 0.9556 | 0.9556 |
| 9 | 2.00E-05 | 32 | 2 | 0.9855 | 0.9667 | 0.9667 |
| 9 | 2.00E-05 | 32 | 3 | 0.9755 | 0.9556 | 0.9556 |
| 9 | 2.00E-05 | 32 | 4 | 0.9820 | 0.9778 | 0.9778 |
| 9 | 2.00E-05 | 32 | 5 | 0.9895 | 0.9554 | 0.9556 |
| 9 | 2.00E-05 | 32 | 6 | 0.9865 | 0.9442 | 0.9444 |
| 9 | 2.00E-05 | 32 | 7 | 0.9930 | 0.8860 | 0.8889 |
| 9 | 2.00E-05 | 32 | 8 | 0.9895 | 0.8860 | 0.8889 |
| 9 | 5.00E-05 | 16 | 1 | 0.9630 | 0.9667 | 0.9667 |
| 9 | 5.00E-05 | 16 | 2 | 0.9910 | 0.9666 | 0.9667 |
| 9 | 5.00E-05 | 16 | 3 | 0.9815 | 0.9666 | 0.9667 |
| 9 | 5.00E-05 | 16 | 4 | 0.9950 | 0.9095 | 0.9111 |
| 9 | 5.00E-05 | 16 | 5 | 0.9915 | 0.9325 | 0.9333 |
| 9 | 5.00E-05 | 16 | 6 | 0.9855 | 0.9778 | 0.9778 |
| 9 | 5.00E-05 | 16 | 7 | 0.9895 | 0.9554 | 0.9556 |
| 9 | 5.00E-05 | 16 | 8 | 0.9795 | 0.9889 | 0.9889 |
| 9 | 5.00E-05 | 32 | 1 | 0.9870 | 0.9105 | 0.9111 |
| 9 | 5.00E-05 | 32 | 2 | 0.9860 | 0.9329 | 0.9333 |
| 9 | 5.00E-05 | 32 | 3 | 0.9875 | 0.9442 | 0.9444 |
| 9 | 5.00E-05 | 32 | 4 | 0.9950 | 0.9889 | 0.9889 |
| 9 | 5.00E-05 | 32 | 5 | 0.9845 | 0.9666 | 0.9667 |
| 9 | 5.00E-05 | 32 | 6 | 0.9865 | 0.9552 | 0.9556 |
| 9 | 5.00E-05 | 32 | 7 | 0.9765 | 0.9095 | 0.9111 |
| 9 | 5.00E-05 | 32 | 8 | 0.9845 | 0.9556 | 0.9556 |
| 10 | 2.00E-05 | 16 | 1 | 0.9990 | 0.9668 | 0.9667 |
| 10 | 2.00E-05 | 16 | 2 | 0.9940 | 0.9331 | 0.9333 |
| 10 | 2.00E-05 | 16 | 3 | 0.9935 | 0.9667 | 0.9667 |
| 10 | 2.00E-05 | 16 | 4 | 0.9930 | 0.9556 | 0.9556 |
| 10 | 2.00E-05 | 16 | 5 | 0.9885 | 0.9556 | 0.9556 |
| 10 | 2.00E-05 | 16 | 6 | 0.9915 | 0.9668 | 0.9667 |
| 10 | 2.00E-05 | 16 | 7 | 0.9930 | 0.9668 | 0.9667 |
| 10 | 2.00E-05 | 16 | 8 | 0.9810 | 0.9556 | 0.9556 |
| 10 | 2.00E-05 | 32 | 1 | 0.9945 | 0.9442 | 0.9444 |
| 10 | 2.00E-05 | 32 | 2 | 0.9965 | 0.9778 | 0.9778 |
| 10 | 2.00E-05 | 32 | 3 | 0.9830 | 0.9444 | 0.9444 |
| 10 | 2.00E-05 | 32 | 4 | 0.9930 | 0.9556 | 0.9556 |
| 10 | 2.00E-05 | 32 | 5 | 0.9880 | 0.9667 | 0.9667 |
| 10 | 2.00E-05 | 32 | 6 | 0.9870 | 0.9778 | 0.9778 |
| 10 | 2.00E-05 | 32 | 7 | 0.9885 | 0.9667 | 0.9667 |
| 10 | 2.00E-05 | 32 | 8 | 0.9840 | 0.9778 | 0.9778 |
| 10 | 5.00E-05 | 16 | 1 | 0.9970 | 0.9778 | 0.9778 |
| 10 | 5.00E-05 | 16 | 2 | 0.9635 | 0.9210 | 0.9222 |
| 10 | 5.00E-05 | 16 | 3 | 0.9940 | 0.9778 | 0.9778 |
| 10 | 5.00E-05 | 16 | 4 | 0.9780 | 0.9556 | 0.9556 |
| 10 | 5.00E-05 | 16 | 5 | 0.9890 | 0.9778 | 0.9778 |
| 10 | 5.00E-05 | 16 | 6 | 0.9825 | 0.9556 | 0.9556 |
| 10 | 5.00E-05 | 16 | 7 | 0.9830 | 0.9668 | 0.9667 |
| 10 | 5.00E-05 | 16 | 8 | 0.9880 | 0.9444 | 0.9444 |
| 10 | 5.00E-05 | 32 | 1 | 0.9920 | 0.9668 | 0.9667 |
| 10 | 5.00E-05 | 32 | 2 | 0.9960 | 0.9442 | 0.9444 |
| 10 | 5.00E-05 | 32 | 3 | 0.9915 | 0.9778 | 0.9778 |
| 10 | 5.00E-05 | 32 | 4 | 0.9905 | 0.9778 | 0.9778 |
| 10 | 5.00E-05 | 32 | 5 | 0.9855 | 0.9668 | 0.9667 |
| 10 | 5.00E-05 | 32 | 6 | 0.9935 | 0.9668 | 0.9667 |
| 10 | 5.00E-05 | 32 | 7 | 0.9880 | 0.9778 | 0.9778 |
| 10 | 5.00E-05 | 32 | 8 | 0.9800 | 0.9778 | 0.9778 |

**Supplementary Table 2c: Validation Statistics for BERT Model on the Holdout Set for Text Relevance Classification**

95% confidence intervals are included in brackets.

|  | **F1** | **Accuracy** | **Precision** | **Recall** | **AUC-ROC** |
| --- | --- | --- | --- | --- | --- |
| **Twitter** | 96.42  (95.65-97.19) | 96.00  (95.13-96.87) | 95.95  (95.02-96.88) | 96.70  (94.76-98.64) | 96.22  (94.25-98.19) |

1. AUC-ROC: Area under the curve for the receiver operating curve.

We selected the candidate epoch number for each platform’s BERT model using 10-fold cross-validation and validated using the holdout set. The final model was selected based on the maximum F1-score, i.e., harmonic mean of precision and recall. Precision and recall are equivalent to positive predictive value and sensitivity respectively.

**Supplementary Table 3: Keywords Identified by Hierarchical Dirichlet Process on General Topic Modelling**

Topics and respective keywords were generated with unsupervised Hierarchical Dirichlet Process (HDP) model. We fused similar topics together and summarised the major areas, subtopics, and topic keywords for each platform. Example tweets were not included since only aggregate data can be presented according to privacy agreement.

| **ID** | **Gross Topic** | **Subtopic** | **Keywords** |
| --- | --- | --- | --- |
| **0** | Awareness | Awareness Campaign for RB | retinoblastoma_care awar_campaign retinoblastoma_awar care_coordin research_retinoblastoma |
| **1** |  | Awareness campaign on common childhood cancers | cancer_day lymphoblast_leukemia myeloid_leukemia retinoblastoma_melanoma genet_counsel |
| **2** |  | Campaigns with Retinoblastoma Survivors Sharing | parent_survivor help_advanc survivor_famili retinoblastoma_survivor thank_share |
| **3** |  | Education on RB Treatment Options | retinoblastoma_treatment ocular_oncolog speak_retinoblastoma enucleation_chemo like_spread |
| **4** |  | One Retinoblastoma World Conference | one_retinoblastoma retinoblastoma_world world_confer oneretinoblastomaworld2020_crrab virtual_confer |
| **5** |  | Retinoblastoma Awareness Week | retinoblastoma_awar awar_week rais_awar affect_retinoblastoma particip_whitathon |
| **6** |  | Urgent need for retinoblastoma care | childhood_cancer need_urgent retinoblastoma_childhood persist_retin associ_retinoblastoma |
| **7** | Patient Support | Financial Support for Chemotherapy | chemo_thank donat_abl help_retinoblastoma share_help get_chemo |
| **8** |  | Financial Support for Prothesis | rare_diseas diseas_retinoblastoma ocular_prosthesi urgent_need velvet_gloves |
| **9** |  | General Fund Raising Campaign | suffer_retinoblastoma need_help cancer_need rais_fund support_perform |
| **10** |  | Patient Support Campaigns | diagnos_bilater everyon_help help_boy pictur_say suffer_retinoblastoma |
| **11** |  | Support to families with retinoblastoma children | retinoblastoma_survivor parent_retinoblastoma survivor_hereditari lost_eye famili_affect |
| **12** |  | Supports to RB survivors | affect_retinoblastoma adult_survivor retinoblastoma_strong diseas_learn share_discuss |
| **13** | Presentation and Diagnosis | Diagnosis of RB with Deep Learning | diagnosi_retinoblastoma retinoblastoma_use deep_learn earli_predict predict_diagnosi |
| **14** |  | Genetic Investigations for Retinoblastoma | tumor_suppressor hereditari_retinoblastoma increas_risk mutat_gene rb_protein |
| **15** |  | Hereditary Retinoblastoma Diagnosis | retinoblastoma_hereditari sign_retinoblastoma case_report earli_detect autosom_domin |
| **16** |  | Home Screening with Smartphone | smartphon_detect detect_eye retinoblastoma_bilater  flash_photo spread_word |
| **17** |  | Images of retinoblastoma survivors | imag_retinoblastoma retinoblastoma_scanner retinoblastoma_survivor rb_survivor review_retinoblastoma |
| **18** |  | Leukocoria Differential Diagnosis | congenit_cataract caus_leukocoria coat_diseas primari_vitreou hyperplast_primari |
| **19** |  | RB Common Clinical Presentation | red_reflex sign_retinoblastoma white_glow leukocoria_white lazi_eye |
| **20** |  | Ways to Incentivise RB Early Diagnosis | spot_soon use_photographi earli_detect tumor_retinoblastoma save_live |
| **21** | Treatment | Alternative Therapy for RB | cancer_cell kill_retinoblastoma ginger_kill herb_retina form_tissu |
| **22** |  | Chemotherapy | childhood_retinoblastoma ocular_chemotherapi pediatr_oncologist retinoblastoma_team team_specialist |
| **23** |  | Effectiveness of IAC in Improving RB Survival ... | intraarteri_chemotherapi eye_rescu treatment_retinoblastoma patient_retinoblastoma eye_salvag |
| **24** |  | Enucleation and Early Surgical Treatment | surgic_remov remov_usual treat_earli prosthet_eye lost_one |
| **25** |  | General Discussions on RB Treatment | treatment_retinoblastoma retinoblastoma_type case_retinoblastoma retinoblastoma_undergo affect_eye |
| **26** |  | Genetics and Pharmacological Treatment for RB | retinoblastoma_treatment risk_retinoblastoma genet_test drug_releas releas_system |
| **27** |  | Immunotherapy for the treatment of retinoblastoma | inject_hydrogel preserv_vision car_cell control_retinoblastoma retinoblastoma_preserv |
| **28** |  | Multidisciplinary approach to Retinoblastoma Treatment | approach_retinoblastoma new_way multidisciplinari_approach treat_retinoblastoma  eye_therapi |
| **29** |  | New Development in RB Treatment | clinic_trial ocular_oncolog retinoblastoma_ocular tumor_appear retinoblastoma_research |
| **30** |  | Oncolytic Virus to Eliminate Tumour Cells | oncolyt_viru viru_attack treatment_retinoblastoma tumor_cell first_time |
| **31** |  | Radiotherapy for RB | patient_treat first_patient linear_acceler therapi_retinoblastoma radiotherapi_retinoblastoma |
| **32** |  | Retinoblastoma treatment unit in Latin America | latin_america first_unit retinoblastoma_latin venezuela_first center_latin |
| **33** |  | Survivors Sharing for Enucleation Experience | year_ago fight_retinoblastoma share_stori decid_remov lost_vision |
| **34** |  | Treatment Service Ease of Access | retinoblastoma_servic retinoblastoma_cost know_us dedic_fund huge_thank |
| **35** |  | Treatment of with Intravitreal Chemotherapy and Enucleation | get_melphalam treatment_retinoblastoma vitreou_seed oncolog_retinoblastoma intravitr_chemotherapi |

Supplementary Table 4: Topics identified by BERTopic

| Topic number | Keywords |
| --- | --- |
| 0 | retinoblastoma, ophthalmologist, specializing, care, rare |
| 1 | retinoblastoma, eyecancer, cáncerocular, cáncer, diagnosticada |
| 2 | retinoblastoma, cancerinfantil, eyecancer, treatment, diagnosis |
| 3 | retinoblastoma, cancer, diagnosed, eyecancer, child |
| 4 | retinoblastoma, _retinoblastoma, câncer, tumor, tumores |
| 5 | retinoblastoma, cancer, neovascular, eye, eyes |
| 6 | retinoblastoma, diagnosed, fundmamalis, clinic, eye |
| 7 | retinoblastoma, retinoblastomaawareness, eyecancer, retina, tumor |
| 8 | retinoblastoma, eye, suffers, daughter, hcastanierj |
| 9 | retinoblastoma, cancer, rare, childhoodcancer, diagnosed |
| 10 | retinoblastoma, childhoodcancer, labuenanoticia, medical, cinthiachavezb |
| 11 | retinoblastoma, retinal, cancer, cataract, eye |
| 12 | retinoblastoma, tumors, tumor, hepatoblastoma, nephroblastoma |
| 13 | retinoblastoma, retinopathy, tumor, pediatroblastos, childhoodcancer |
| 14 | retinoblastoma, cancer, rare, diagnosed, eye |
| 15 | retinoblastoma, cáncer, co, extirpar, ojo |
| 16 | retinoblastoma, cancer, eye, childhoodcancer, photography |
| 17 | retinoblastoma, retinal, retina, tumors, tumor |
| 18 | retinoblastoma, eye, prosthetic, cyanide, lrjevbwtea |
| 19 | retinoblastoma, eye, cancer, diagnosed, impacientesvzla |
| 20 | retinoblastoma, cataracts, eye, patients, diagnosed |
| 21 | retinoblastoma, treatment, propeñarol, retweeted, propeniarol |
| 22 | retinoblastoma, retinalphys, ophthalmic, ocular, intravitreal |
| 23 | retinoblastoma, surgical, eye, operation, bilateral |
| 24 | retinoblastoma, ocular, treatment, tumor, recuerdo |
| 25 | retinoblastoma, prosthesisoculars, eyes, hospital, cancer |
| 26 | retinoblastoma, glioblastoma, tumor, inhibitor, therapeutic |
| 27 | retinoblastoma, cataracts, eye, toxoplasmosis, examination |
| 28 | retinoblastoma, eye, treatment, bilateral, eyes |
| 29 | retinoblastoma, recaudado, luz, cancerinfantil, urgente |
| 30 | retinoblastoma, tumor, tecnología, pediatrics, diagnosis |
| 31 | retinoblastoma, cancer, oncology, sjdbarcelona_es, oncolytic |
| 32 | retinoblastoma, tumors, cancer, cáncerinfantil, cancers |
| 33 | retinoblastoma, joelisana, erikavelezz, desperate, girl |
| 34 | retinoblastoma, elojitodelalito, cancer, quimioterapia, hijo |
| 35 | retinoblastoma, retinaserv, retina, tumor, treatment |
| 36 | retinoblastoma, santiago, mariangel, rodríguez, luzdeanael_ya |
| 37 | retinoblastoma, juan, eyetreatment, eyeclinicinlagos, eyeclinicinabuja |
| 38 | retinoblastoma, mcdc_mcdc, mz1npmgl9u, seneedec, rt |
| 39 | retinoblastoma, fundmamalis, childhoodcancer, diagnosed, oncology |
| 40 | retinoblastoma, jeimmy, treatment, rt, médi |
| 41 | retinoblastoma, diagnosed, treatment, oncology, cancer |

**Supplementary Table 5: Overall Cross-validation Statistics for BERT Model for Text Sentiment Classification**

**Supplementary Table 5a: Overall Cross-validation Statistics for BERT Model for Text Sentiment Classification**

| **Learning Rate** | **Batch Size** | **Epochs** | **AUC** | **F1 Score** | **Accuracy** |
| --- | --- | --- | --- | --- | --- |
| **2E-05** | 16 | 1 | 0.724 [0.691-0.758] | 0.505 [0.462-0.549] | 54.4% [51.2%-57.7%] |
| **2E-05** | 16 | 2 | 0.819 [0.806-0.832] | 0.621 [0.584-0.658] | 64.2% [61.5%-66.9%] |
| **2E-05** | 16 | 3 | 0.839 [0.830-0.848] | 0.684 [0.671-0.697] | 68.9% [67.6%-70.2%] |
| **2E-05** | 16 | 4 | 0.845 [0.834-0.857] | 0.694 [0.675-0.712] | 69.7% [68.0%-71.4%] |
| **2E-05** | 16 | 5 | 0.841 [0.825-0.857] | 0.676 [0.656-0.697] | 68.1% [66.1%-70.1%] |
| **2E-05** | 16 | 6 | 0.836 [0.824-0.848] | 0.681 [0.661-0.701] | 68.6% [66.8%-70.4%] |
| **2E-05** | 16 | 7 | 0.822 [0.804-0.840] | 0.657 [0.626-0.687] | 65.8% [62.6%-69.0%] |
| **2E-05** | 16 | 8 | 0.842 [0.831-0.853] | 0.687 [0.671-0.704] | 69.3% [67.6%-71.0%] |
| **2E-05** | 32 | 1 | 0.695 [0.672-0.719] | 0.450 [0.424-0.476] | 50.1% [47.7%-52.5%] |
| **2E-05** | 32 | 2 | 0.768 [0.748-0.788] | 0.582 [0.549-0.616] | 61.1% [58.0%-64.1%] |
| **2E-05** | 32 | 3 | 0.811 [0.794-0.829] | 0.646 [0.612-0.680] | 65.5% [62.5%-68.5%] |
| **2E-05** | 32 | 4 | 0.824 [0.808-0.840] | 0.646 [0.612-0.680] | 65.5% [62.4%-68.6%] |
| **2E-05** | 32 | 5 | 0.827 [0.817-0.838] | 0.659 [0.645-0.674] | 66.6% [65.4%-67.8%] |
| **2E-05** | 32 | 6 | 0.828 [0.814-0.842] | 0.671 [0.657-0.685] | 67.2% [65.8%-68.7%] |
| **2E-05** | 32 | 7 | 0.833 [0.818-0.848] | 0.673 [0.651-0.695] | 67.7% [65.5%-69.9%] |
| **2E-05** | 32 | 8 | 0.826 [0.811-0.841] | 0.669 [0.647-0.691] | 67.2% [65.2%-69.3%] |
| **5E-05** | 16 | 1 | 0.782 [0.751-0.814] | 0.571 [0.531-0.611] | 60.8% [57.3%-64.2%] |
| **5E-05** | 16 | 2 | 0.861 [0.844-0.879] | 0.708 [0.677-0.738] | 71.4% [68.5%-74.2%] |
| **5E-05** | 16 | 3 | 0.860 [0.843-0.876] | 0.689 [0.669-0.710] | 69.6% [67.7%-71.5%] |
| **5E-05** | 16 | 4 | 0.855 [0.840-0.870] | 0.704 [0.679-0.729] | 71.1% [69.0%-73.3%] |
| **5E-05** | 16 | 5 | 0.852 [0.837-0.867] | 0.703 [0.677-0.728] | 70.7% [68.2%-73.1%] |
| **5E-05** | 16 | 6 | 0.842 [0.829-0.856] | 0.710 [0.687-0.732] | 71.2% [69.0%-73.4%] |
| **5E-05** | 16 | 7 | 0.848 [0.832-0.863] | 0.697 [0.670-0.725] | 70.3% [68.0%-72.7%] |
| **5E-05** | 16 | 8 | 0.852 [0.837-0.868] | 0.713 [0.694-0.733] | 71.8% [70.0%-73.5%] |
| **5E-05** | 32 | 1 | 0.758 [0.730-0.785] | 0.547 [0.487-0.606] | 58.3% [54.0%-62.6%] |
| **5E-05** | 32 | 2 | 0.821 [0.801-0.842] | 0.645 [0.609-0.680] | 66.3% [63.4%-69.2%] |
| **5E-05** | 32 | 3 | 0.852 [0.842-0.862] | 0.686 [0.658-0.714] | 69.6% [67.4%-71.8%] |
| **5E-05** | 32 | 4 | 0.848 [0.833-0.863] | 0.703 [0.680-0.725] | 70.5% [68.1%-72.8%] |
| **5E-05** | 32 | 5 | 0.841 [0.831-0.852] | 0.673 [0.648-0.698] | 67.7% [65.6%-69.8%] |
| **5E-05** | 32 | 6 | 0.836 [0.824-0.847] | 0.696 [0.683-0.709] | 69.8% [68.8%-70.9%] |
| **5E-05** | 32 | 7 | 0.832 [0.820-0.844] | 0.682 [0.667-0.696] | 68.5% [67.1%-69.8%] |
| **5E-05** | 32 | 8 | 0.841 [0.825-0.857] | 0.690 [0.659-0.721] | 69.3% [66.3%-72.4%] |

**Supplementary Table 5b: Per-fold Cross-validation Statistics for BERT Model for Text Sentiment Classification**

| Fold | Learning Rate | Batch Size | Epochs | AUC | F1 Score | Accuracy |
| --- | --- | --- | --- | --- | --- | --- |
| 1 | 2.00E-05 | 16 | 1 | 0.6656 | 0.4466 | 0.5050 |
| 1 | 2.00E-05 | 16 | 2 | 0.8162 | 0.6322 | 0.6450 |
| 1 | 2.00E-05 | 16 | 3 | 0.8296 | 0.6789 | 0.6800 |
| 1 | 2.00E-05 | 16 | 4 | 0.8584 | 0.6912 | 0.7000 |
| 1 | 2.00E-05 | 16 | 5 | 0.8510 | 0.6896 | 0.6900 |
| 1 | 2.00E-05 | 16 | 6 | 0.8151 | 0.6467 | 0.6450 |
| 1 | 2.00E-05 | 16 | 7 | 0.8471 | 0.6949 | 0.7000 |
| 1 | 2.00E-05 | 16 | 8 | 0.8537 | 0.7098 | 0.7100 |
| 1 | 2.00E-05 | 32 | 1 | 0.6312 | 0.4061 | 0.4500 |
| 1 | 2.00E-05 | 32 | 2 | 0.7409 | 0.5465 | 0.5600 |
| 1 | 2.00E-05 | 32 | 3 | 0.7784 | 0.6060 | 0.6100 |
| 1 | 2.00E-05 | 32 | 4 | 0.8140 | 0.6338 | 0.6400 |
| 1 | 2.00E-05 | 32 | 5 | 0.8296 | 0.6785 | 0.6850 |
| 1 | 2.00E-05 | 32 | 6 | 0.8222 | 0.6632 | 0.6550 |
| 1 | 2.00E-05 | 32 | 7 | 0.8579 | 0.7092 | 0.7150 |
| 1 | 2.00E-05 | 32 | 8 | 0.8183 | 0.6804 | 0.6800 |
| 1 | 5.00E-05 | 16 | 1 | 0.7396 | 0.5625 | 0.6000 |
| 1 | 5.00E-05 | 16 | 2 | 0.8654 | 0.7462 | 0.7500 |
| 1 | 5.00E-05 | 16 | 3 | 0.8488 | 0.6653 | 0.6700 |
| 1 | 5.00E-05 | 16 | 4 | 0.8694 | 0.6987 | 0.7000 |
| 1 | 5.00E-05 | 16 | 5 | 0.8131 | 0.6181 | 0.6250 |
| 1 | 5.00E-05 | 16 | 6 | 0.8275 | 0.7151 | 0.7200 |
| 1 | 5.00E-05 | 16 | 7 | 0.8433 | 0.7205 | 0.7200 |
| 1 | 5.00E-05 | 16 | 8 | 0.8330 | 0.6986 | 0.7100 |
| 1 | 5.00E-05 | 32 | 1 | 0.7070 | 0.5423 | 0.5550 |
| 1 | 5.00E-05 | 32 | 2 | 0.7496 | 0.5308 | 0.5750 |
| 1 | 5.00E-05 | 32 | 3 | 0.8550 | 0.7176 | 0.7250 |
| 1 | 5.00E-05 | 32 | 4 | 0.8613 | 0.7229 | 0.7300 |
| 1 | 5.00E-05 | 32 | 5 | 0.8382 | 0.6700 | 0.6600 |
| 1 | 5.00E-05 | 32 | 6 | 0.8434 | 0.6909 | 0.6850 |
| 1 | 5.00E-05 | 32 | 7 | 0.8460 | 0.6915 | 0.6900 |
| 1 | 5.00E-05 | 32 | 8 | 0.8332 | 0.6914 | 0.7000 |
| 2 | 2.00E-05 | 16 | 1 | 0.7281 | 0.4990 | 0.5600 |
| 2 | 2.00E-05 | 16 | 2 | 0.8285 | 0.5317 | 0.5850 |
| 2 | 2.00E-05 | 16 | 3 | 0.8486 | 0.6989 | 0.7100 |
| 2 | 2.00E-05 | 16 | 4 | 0.8542 | 0.7119 | 0.7150 |
| 2 | 2.00E-05 | 16 | 5 | 0.8534 | 0.6742 | 0.6700 |
| 2 | 2.00E-05 | 16 | 6 | 0.8488 | 0.7030 | 0.7050 |
| 2 | 2.00E-05 | 16 | 7 | 0.8309 | 0.6844 | 0.6850 |
| 2 | 2.00E-05 | 16 | 8 | 0.8348 | 0.6867 | 0.7000 |
| 2 | 2.00E-05 | 32 | 1 | 0.7223 | 0.4381 | 0.5100 |
| 2 | 2.00E-05 | 32 | 2 | 0.7572 | 0.5876 | 0.6350 |
| 2 | 2.00E-05 | 32 | 3 | 0.8141 | 0.6555 | 0.6550 |
| 2 | 2.00E-05 | 32 | 4 | 0.8303 | 0.5837 | 0.6150 |
| 2 | 2.00E-05 | 32 | 5 | 0.8176 | 0.6408 | 0.6500 |
| 2 | 2.00E-05 | 32 | 6 | 0.8495 | 0.6828 | 0.6850 |
| 2 | 2.00E-05 | 32 | 7 | 0.8384 | 0.7000 | 0.7050 |
| 2 | 2.00E-05 | 32 | 8 | 0.8677 | 0.7043 | 0.7200 |
| 2 | 5.00E-05 | 16 | 1 | 0.8002 | 0.5368 | 0.6000 |
| 2 | 5.00E-05 | 16 | 2 | 0.8562 | 0.6908 | 0.6900 |
| 2 | 5.00E-05 | 16 | 3 | 0.8730 | 0.7134 | 0.7100 |
| 2 | 5.00E-05 | 16 | 4 | 0.8651 | 0.6677 | 0.6900 |
| 2 | 5.00E-05 | 16 | 5 | 0.8568 | 0.7047 | 0.7050 |
| 2 | 5.00E-05 | 16 | 6 | 0.8618 | 0.7169 | 0.7100 |
| 2 | 5.00E-05 | 16 | 7 | 0.8355 | 0.6602 | 0.6900 |
| 2 | 5.00E-05 | 16 | 8 | 0.8533 | 0.7342 | 0.7350 |
| 2 | 5.00E-05 | 32 | 1 | 0.7931 | 0.6172 | 0.6400 |
| 2 | 5.00E-05 | 32 | 2 | 0.8224 | 0.6050 | 0.6150 |
| 2 | 5.00E-05 | 32 | 3 | 0.8513 | 0.6888 | 0.6850 |
| 2 | 5.00E-05 | 32 | 4 | 0.8154 | 0.6555 | 0.6600 |
| 2 | 5.00E-05 | 32 | 5 | 0.8591 | 0.6965 | 0.6950 |
| 2 | 5.00E-05 | 32 | 6 | 0.8427 | 0.7060 | 0.7050 |
| 2 | 5.00E-05 | 32 | 7 | 0.8500 | 0.6895 | 0.6850 |
| 2 | 5.00E-05 | 32 | 8 | 0.8680 | 0.7037 | 0.7000 |
| 3 | 2.00E-05 | 16 | 1 | 0.7411 | 0.4584 | 0.5050 |
| 3 | 2.00E-05 | 16 | 2 | 0.8510 | 0.6913 | 0.7000 |
| 3 | 2.00E-05 | 16 | 3 | 0.8434 | 0.6722 | 0.6800 |
| 3 | 2.00E-05 | 16 | 4 | 0.8531 | 0.7301 | 0.7300 |
| 3 | 2.00E-05 | 16 | 5 | 0.8510 | 0.7145 | 0.7150 |
| 3 | 2.00E-05 | 16 | 6 | 0.8552 | 0.7009 | 0.7000 |
| 3 | 2.00E-05 | 16 | 7 | 0.8528 | 0.7075 | 0.7100 |
| 3 | 2.00E-05 | 16 | 8 | 0.8363 | 0.6796 | 0.6850 |
| 3 | 2.00E-05 | 32 | 1 | 0.7357 | 0.4644 | 0.5150 |
| 3 | 2.00E-05 | 32 | 2 | 0.8099 | 0.6456 | 0.6550 |
| 3 | 2.00E-05 | 32 | 3 | 0.8105 | 0.6779 | 0.6850 |
| 3 | 2.00E-05 | 32 | 4 | 0.8413 | 0.7057 | 0.7050 |
| 3 | 2.00E-05 | 32 | 5 | 0.8480 | 0.6849 | 0.6850 |
| 3 | 2.00E-05 | 32 | 6 | 0.8287 | 0.6855 | 0.6850 |
| 3 | 2.00E-05 | 32 | 7 | 0.8449 | 0.6844 | 0.6850 |
| 3 | 2.00E-05 | 32 | 8 | 0.8275 | 0.6760 | 0.6850 |
| 3 | 5.00E-05 | 16 | 1 | 0.8058 | 0.5706 | 0.6000 |
| 3 | 5.00E-05 | 16 | 2 | 0.8207 | 0.6451 | 0.6500 |
| 3 | 5.00E-05 | 16 | 3 | 0.8720 | 0.7312 | 0.7350 |
| 3 | 5.00E-05 | 16 | 4 | 0.8738 | 0.7246 | 0.7250 |
| 3 | 5.00E-05 | 16 | 5 | 0.8886 | 0.7384 | 0.7450 |
| 3 | 5.00E-05 | 16 | 6 | 0.8594 | 0.7320 | 0.7400 |
| 3 | 5.00E-05 | 16 | 7 | 0.8760 | 0.7388 | 0.7450 |
| 3 | 5.00E-05 | 16 | 8 | 0.8708 | 0.7265 | 0.7350 |
| 3 | 5.00E-05 | 32 | 1 | 0.7478 | 0.4129 | 0.5050 |
| 3 | 5.00E-05 | 32 | 2 | 0.8443 | 0.6342 | 0.6750 |
| 3 | 5.00E-05 | 32 | 3 | 0.8581 | 0.6820 | 0.6950 |
| 3 | 5.00E-05 | 32 | 4 | 0.8262 | 0.6713 | 0.6700 |
| 3 | 5.00E-05 | 32 | 5 | 0.8452 | 0.7126 | 0.7150 |
| 3 | 5.00E-05 | 32 | 6 | 0.8525 | 0.6949 | 0.7000 |
| 3 | 5.00E-05 | 32 | 7 | 0.8292 | 0.6800 | 0.6750 |
| 3 | 5.00E-05 | 32 | 8 | 0.8516 | 0.7011 | 0.6950 |
| 4 | 2.00E-05 | 16 | 1 | 0.6918 | 0.5107 | 0.5500 |
| 4 | 2.00E-05 | 16 | 2 | 0.8141 | 0.5754 | 0.6000 |
| 4 | 2.00E-05 | 16 | 3 | 0.8443 | 0.6717 | 0.6750 |
| 4 | 2.00E-05 | 16 | 4 | 0.8467 | 0.6809 | 0.6800 |
| 4 | 2.00E-05 | 16 | 5 | 0.8414 | 0.6489 | 0.6550 |
| 4 | 2.00E-05 | 16 | 6 | 0.8489 | 0.7241 | 0.7250 |
| 4 | 2.00E-05 | 16 | 7 | 0.8284 | 0.6599 | 0.6450 |
| 4 | 2.00E-05 | 16 | 8 | 0.8440 | 0.6724 | 0.6750 |
| 4 | 2.00E-05 | 32 | 1 | 0.6979 | 0.4623 | 0.5100 |
| 4 | 2.00E-05 | 32 | 2 | 0.7642 | 0.6395 | 0.6650 |
| 4 | 2.00E-05 | 32 | 3 | 0.8226 | 0.6927 | 0.6950 |
| 4 | 2.00E-05 | 32 | 4 | 0.8352 | 0.6847 | 0.6950 |
| 4 | 2.00E-05 | 32 | 5 | 0.8313 | 0.6833 | 0.6900 |
| 4 | 2.00E-05 | 32 | 6 | 0.8354 | 0.6792 | 0.6800 |
| 4 | 2.00E-05 | 32 | 7 | 0.8388 | 0.6657 | 0.6800 |
| 4 | 2.00E-05 | 32 | 8 | 0.8361 | 0.7118 | 0.7100 |
| 4 | 5.00E-05 | 16 | 1 | 0.8025 | 0.5950 | 0.6300 |
| 4 | 5.00E-05 | 16 | 2 | 0.8749 | 0.7223 | 0.7300 |
| 4 | 5.00E-05 | 16 | 3 | 0.8628 | 0.6872 | 0.7000 |
| 4 | 5.00E-05 | 16 | 4 | 0.8580 | 0.7120 | 0.7200 |
| 4 | 5.00E-05 | 16 | 5 | 0.8548 | 0.7146 | 0.7150 |
| 4 | 5.00E-05 | 16 | 6 | 0.8301 | 0.6978 | 0.6950 |
| 4 | 5.00E-05 | 16 | 7 | 0.8676 | 0.7257 | 0.7200 |
| 4 | 5.00E-05 | 16 | 8 | 0.8784 | 0.7618 | 0.7600 |
| 4 | 5.00E-05 | 32 | 1 | 0.8119 | 0.6602 | 0.6800 |
| 4 | 5.00E-05 | 32 | 2 | 0.8133 | 0.6616 | 0.6800 |
| 4 | 5.00E-05 | 32 | 3 | 0.8653 | 0.7136 | 0.7150 |
| 4 | 5.00E-05 | 32 | 4 | 0.8497 | 0.6880 | 0.6850 |
| 4 | 5.00E-05 | 32 | 5 | 0.8583 | 0.7058 | 0.7050 |
| 4 | 5.00E-05 | 32 | 6 | 0.8515 | 0.7256 | 0.7250 |
| 4 | 5.00E-05 | 32 | 7 | 0.8084 | 0.6775 | 0.6900 |
| 4 | 5.00E-05 | 32 | 8 | 0.8142 | 0.6413 | 0.6350 |
| 5 | 2.00E-05 | 16 | 1 | 0.7715 | 0.5655 | 0.5750 |
| 5 | 2.00E-05 | 16 | 2 | 0.8168 | 0.6149 | 0.6500 |
| 5 | 2.00E-05 | 16 | 3 | 0.8606 | 0.7276 | 0.7300 |
| 5 | 2.00E-05 | 16 | 4 | 0.8652 | 0.7289 | 0.7300 |
| 5 | 2.00E-05 | 16 | 5 | 0.8327 | 0.6800 | 0.6850 |
| 5 | 2.00E-05 | 16 | 6 | 0.8413 | 0.7065 | 0.7100 |
| 5 | 2.00E-05 | 16 | 7 | 0.7947 | 0.6102 | 0.6100 |
| 5 | 2.00E-05 | 16 | 8 | 0.8445 | 0.7232 | 0.7300 |
| 5 | 2.00E-05 | 32 | 1 | 0.6725 | 0.3800 | 0.4450 |
| 5 | 2.00E-05 | 32 | 2 | 0.7987 | 0.6232 | 0.6250 |
| 5 | 2.00E-05 | 32 | 3 | 0.8439 | 0.7015 | 0.7050 |
| 5 | 2.00E-05 | 32 | 4 | 0.8023 | 0.6196 | 0.6350 |
| 5 | 2.00E-05 | 32 | 5 | 0.8375 | 0.6695 | 0.6650 |
| 5 | 2.00E-05 | 32 | 6 | 0.8388 | 0.6839 | 0.6900 |
| 5 | 2.00E-05 | 32 | 7 | 0.8628 | 0.7111 | 0.7000 |
| 5 | 2.00E-05 | 32 | 8 | 0.8237 | 0.6911 | 0.6850 |
| 5 | 5.00E-05 | 16 | 1 | 0.7860 | 0.6028 | 0.6150 |
| 5 | 5.00E-05 | 16 | 2 | 0.8927 | 0.7573 | 0.7550 |
| 5 | 5.00E-05 | 16 | 3 | 0.8638 | 0.6668 | 0.6800 |
| 5 | 5.00E-05 | 16 | 4 | 0.8510 | 0.7296 | 0.7350 |
| 5 | 5.00E-05 | 16 | 5 | 0.8344 | 0.6974 | 0.7050 |
| 5 | 5.00E-05 | 16 | 6 | 0.8324 | 0.6925 | 0.6950 |
| 5 | 5.00E-05 | 16 | 7 | 0.8624 | 0.7419 | 0.7400 |
| 5 | 5.00E-05 | 16 | 8 | 0.8212 | 0.6989 | 0.7050 |
| 5 | 5.00E-05 | 32 | 1 | 0.7734 | 0.5503 | 0.5750 |
| 5 | 5.00E-05 | 32 | 2 | 0.8427 | 0.7141 | 0.7150 |
| 5 | 5.00E-05 | 32 | 3 | 0.8329 | 0.6611 | 0.6800 |
| 5 | 5.00E-05 | 32 | 4 | 0.8539 | 0.7418 | 0.7450 |
| 5 | 5.00E-05 | 32 | 5 | 0.8467 | 0.6396 | 0.6550 |
| 5 | 5.00E-05 | 32 | 6 | 0.8102 | 0.6772 | 0.6900 |
| 5 | 5.00E-05 | 32 | 7 | 0.8105 | 0.6502 | 0.6550 |
| 5 | 5.00E-05 | 32 | 8 | 0.8513 | 0.7335 | 0.7350 |
| 6 | 2.00E-05 | 16 | 1 | 0.7742 | 0.5378 | 0.5650 |
| 6 | 2.00E-05 | 16 | 2 | 0.8169 | 0.6854 | 0.6800 |
| 6 | 2.00E-05 | 16 | 3 | 0.8452 | 0.6781 | 0.6900 |
| 6 | 2.00E-05 | 16 | 4 | 0.8496 | 0.6709 | 0.6800 |
| 6 | 2.00E-05 | 16 | 5 | 0.8372 | 0.6627 | 0.6800 |
| 6 | 2.00E-05 | 16 | 6 | 0.8482 | 0.6889 | 0.6950 |
| 6 | 2.00E-05 | 16 | 7 | 0.8162 | 0.6407 | 0.6450 |
| 6 | 2.00E-05 | 16 | 8 | 0.8357 | 0.6886 | 0.7050 |
| 6 | 2.00E-05 | 32 | 1 | 0.6865 | 0.4433 | 0.5100 |
| 6 | 2.00E-05 | 32 | 2 | 0.7799 | 0.6009 | 0.6400 |
| 6 | 2.00E-05 | 32 | 3 | 0.7871 | 0.5754 | 0.5900 |
| 6 | 2.00E-05 | 32 | 4 | 0.8524 | 0.6476 | 0.6500 |
| 6 | 2.00E-05 | 32 | 5 | 0.8429 | 0.6297 | 0.6450 |
| 6 | 2.00E-05 | 32 | 6 | 0.8367 | 0.6843 | 0.6850 |
| 6 | 2.00E-05 | 32 | 7 | 0.8282 | 0.6526 | 0.6650 |
| 6 | 2.00E-05 | 32 | 8 | 0.7967 | 0.6105 | 0.6300 |
| 6 | 5.00E-05 | 16 | 1 | 0.8022 | 0.6075 | 0.6650 |
| 6 | 5.00E-05 | 16 | 2 | 0.8472 | 0.6849 | 0.7000 |
| 6 | 5.00E-05 | 16 | 3 | 0.8774 | 0.6765 | 0.6900 |
| 6 | 5.00E-05 | 16 | 4 | 0.8670 | 0.7079 | 0.7150 |
| 6 | 5.00E-05 | 16 | 5 | 0.8466 | 0.6848 | 0.6950 |
| 6 | 5.00E-05 | 16 | 6 | 0.8087 | 0.6567 | 0.6600 |
| 6 | 5.00E-05 | 16 | 7 | 0.8178 | 0.6971 | 0.7000 |
| 6 | 5.00E-05 | 16 | 8 | 0.8623 | 0.7009 | 0.7000 |
| 6 | 5.00E-05 | 32 | 1 | 0.7310 | 0.4195 | 0.5100 |
| 6 | 5.00E-05 | 32 | 2 | 0.8213 | 0.6459 | 0.6700 |
| 6 | 5.00E-05 | 32 | 3 | 0.8419 | 0.6838 | 0.6950 |
| 6 | 5.00E-05 | 32 | 4 | 0.8449 | 0.6962 | 0.7050 |
| 6 | 5.00E-05 | 32 | 5 | 0.8262 | 0.6277 | 0.6400 |
| 6 | 5.00E-05 | 32 | 6 | 0.8220 | 0.6824 | 0.6950 |
| 6 | 5.00E-05 | 32 | 7 | 0.8345 | 0.7001 | 0.7050 |
| 6 | 5.00E-05 | 32 | 8 | 0.8254 | 0.6653 | 0.6850 |
| 7 | 2.00E-05 | 16 | 1 | 0.7885 | 0.6389 | 0.6400 |
| 7 | 2.00E-05 | 16 | 2 | 0.8157 | 0.5733 | 0.6300 |
| 7 | 2.00E-05 | 16 | 3 | 0.8373 | 0.6904 | 0.6950 |
| 7 | 2.00E-05 | 16 | 4 | 0.8321 | 0.6968 | 0.6950 |
| 7 | 2.00E-05 | 16 | 5 | 0.8539 | 0.7079 | 0.7150 |
| 7 | 2.00E-05 | 16 | 6 | 0.8212 | 0.6732 | 0.6800 |
| 7 | 2.00E-05 | 16 | 7 | 0.8323 | 0.6885 | 0.6950 |
| 7 | 2.00E-05 | 16 | 8 | 0.8492 | 0.7043 | 0.7100 |
| 7 | 2.00E-05 | 32 | 1 | 0.7409 | 0.5115 | 0.5550 |
| 7 | 2.00E-05 | 32 | 2 | 0.7735 | 0.5241 | 0.5750 |
| 7 | 2.00E-05 | 32 | 3 | 0.8288 | 0.7023 | 0.7050 |
| 7 | 2.00E-05 | 32 | 4 | 0.8211 | 0.6551 | 0.6600 |
| 7 | 2.00E-05 | 32 | 5 | 0.8226 | 0.6668 | 0.6700 |
| 7 | 2.00E-05 | 32 | 6 | 0.8301 | 0.6793 | 0.6850 |
| 7 | 2.00E-05 | 32 | 7 | 0.8165 | 0.6715 | 0.6750 |
| 7 | 2.00E-05 | 32 | 8 | 0.8303 | 0.6510 | 0.6550 |
| 7 | 5.00E-05 | 16 | 1 | 0.8181 | 0.6227 | 0.6550 |
| 7 | 5.00E-05 | 16 | 2 | 0.8487 | 0.6777 | 0.7000 |
| 7 | 5.00E-05 | 16 | 3 | 0.8415 | 0.6756 | 0.6850 |
| 7 | 5.00E-05 | 16 | 4 | 0.8301 | 0.7191 | 0.7250 |
| 7 | 5.00E-05 | 16 | 5 | 0.8466 | 0.6951 | 0.6950 |
| 7 | 5.00E-05 | 16 | 6 | 0.8534 | 0.7467 | 0.7450 |
| 7 | 5.00E-05 | 16 | 7 | 0.8505 | 0.6713 | 0.6750 |
| 7 | 5.00E-05 | 16 | 8 | 0.8363 | 0.7264 | 0.7250 |
| 7 | 5.00E-05 | 32 | 1 | 0.7915 | 0.5572 | 0.6050 |
| 7 | 5.00E-05 | 32 | 2 | 0.8294 | 0.6860 | 0.7000 |
| 7 | 5.00E-05 | 32 | 3 | 0.8540 | 0.7376 | 0.7400 |
| 7 | 5.00E-05 | 32 | 4 | 0.8574 | 0.7393 | 0.7400 |
| 7 | 5.00E-05 | 32 | 5 | 0.8437 | 0.7149 | 0.7150 |
| 7 | 5.00E-05 | 32 | 6 | 0.8269 | 0.6781 | 0.6750 |
| 7 | 5.00E-05 | 32 | 7 | 0.8379 | 0.7042 | 0.7100 |
| 7 | 5.00E-05 | 32 | 8 | 0.8036 | 0.6021 | 0.6100 |
| 8 | 2.00E-05 | 16 | 1 | 0.7393 | 0.4869 | 0.5500 |
| 8 | 2.00E-05 | 16 | 2 | 0.8351 | 0.6466 | 0.6500 |
| 8 | 2.00E-05 | 16 | 3 | 0.8370 | 0.6856 | 0.6800 |
| 8 | 2.00E-05 | 16 | 4 | 0.8519 | 0.7057 | 0.7050 |
| 8 | 2.00E-05 | 16 | 5 | 0.8763 | 0.7052 | 0.7100 |
| 8 | 2.00E-05 | 16 | 6 | 0.8474 | 0.6495 | 0.6750 |
| 8 | 2.00E-05 | 16 | 7 | 0.8337 | 0.6499 | 0.6600 |
| 8 | 2.00E-05 | 16 | 8 | 0.8735 | 0.7020 | 0.7000 |
| 8 | 2.00E-05 | 32 | 1 | 0.7062 | 0.4663 | 0.5350 |
| 8 | 2.00E-05 | 32 | 2 | 0.7666 | 0.5412 | 0.6000 |
| 8 | 2.00E-05 | 32 | 3 | 0.8410 | 0.6439 | 0.6550 |
| 8 | 2.00E-05 | 32 | 4 | 0.8553 | 0.7243 | 0.7250 |
| 8 | 2.00E-05 | 32 | 5 | 0.8309 | 0.6580 | 0.6700 |
| 8 | 2.00E-05 | 32 | 6 | 0.8485 | 0.6824 | 0.6850 |
| 8 | 2.00E-05 | 32 | 7 | 0.8360 | 0.6853 | 0.6900 |
| 8 | 2.00E-05 | 32 | 8 | 0.8464 | 0.6703 | 0.6650 |
| 8 | 5.00E-05 | 16 | 1 | 0.8382 | 0.6180 | 0.6400 |
| 8 | 5.00E-05 | 16 | 2 | 0.9049 | 0.7793 | 0.7800 |
| 8 | 5.00E-05 | 16 | 3 | 0.8803 | 0.7060 | 0.7100 |
| 8 | 5.00E-05 | 16 | 4 | 0.8371 | 0.6968 | 0.7050 |
| 8 | 5.00E-05 | 16 | 5 | 0.8634 | 0.7128 | 0.7200 |
| 8 | 5.00E-05 | 16 | 6 | 0.8686 | 0.7523 | 0.7550 |
| 8 | 5.00E-05 | 16 | 7 | 0.8732 | 0.7181 | 0.7250 |
| 8 | 5.00E-05 | 16 | 8 | 0.8799 | 0.7283 | 0.7300 |
| 8 | 5.00E-05 | 32 | 1 | 0.7551 | 0.6039 | 0.6200 |
| 8 | 5.00E-05 | 32 | 2 | 0.8468 | 0.6664 | 0.6750 |
| 8 | 5.00E-05 | 32 | 3 | 0.8632 | 0.6432 | 0.6650 |
| 8 | 5.00E-05 | 32 | 4 | 0.8885 | 0.7296 | 0.7300 |
| 8 | 5.00E-05 | 32 | 5 | 0.8427 | 0.6395 | 0.6600 |
| 8 | 5.00E-05 | 32 | 6 | 0.8571 | 0.7250 | 0.7200 |
| 8 | 5.00E-05 | 32 | 7 | 0.8571 | 0.7042 | 0.7000 |
| 8 | 5.00E-05 | 32 | 8 | 0.8624 | 0.7415 | 0.7500 |
| 9 | 2.00E-05 | 16 | 1 | 0.6587 | 0.4612 | 0.5150 |
| 9 | 2.00E-05 | 16 | 2 | 0.8148 | 0.6572 | 0.6750 |
| 9 | 2.00E-05 | 16 | 3 | 0.8176 | 0.6680 | 0.6750 |
| 9 | 2.00E-05 | 16 | 4 | 0.8272 | 0.6487 | 0.6600 |
| 9 | 2.00E-05 | 16 | 5 | 0.7951 | 0.6254 | 0.6300 |
| 9 | 2.00E-05 | 16 | 6 | 0.8183 | 0.6649 | 0.6750 |
| 9 | 2.00E-05 | 16 | 7 | 0.7659 | 0.5654 | 0.5650 |
| 9 | 2.00E-05 | 16 | 8 | 0.8171 | 0.6489 | 0.6550 |
| 9 | 2.00E-05 | 32 | 1 | 0.6778 | 0.4602 | 0.4850 |
| 9 | 2.00E-05 | 32 | 2 | 0.7746 | 0.5984 | 0.6150 |
| 9 | 2.00E-05 | 32 | 3 | 0.8114 | 0.5954 | 0.6300 |
| 9 | 2.00E-05 | 32 | 4 | 0.8039 | 0.6250 | 0.6400 |
| 9 | 2.00E-05 | 32 | 5 | 0.8016 | 0.6486 | 0.6600 |
| 9 | 2.00E-05 | 32 | 6 | 0.7872 | 0.6393 | 0.6400 |
| 9 | 2.00E-05 | 32 | 7 | 0.7998 | 0.6194 | 0.6200 |
| 9 | 2.00E-05 | 32 | 8 | 0.8098 | 0.6481 | 0.6450 |
| 9 | 5.00E-05 | 16 | 1 | 0.7046 | 0.4327 | 0.4950 |
| 9 | 5.00E-05 | 16 | 2 | 0.8531 | 0.6682 | 0.6750 |
| 9 | 5.00E-05 | 16 | 3 | 0.8040 | 0.6460 | 0.6500 |
| 9 | 5.00E-05 | 16 | 4 | 0.8181 | 0.6302 | 0.6450 |
| 9 | 5.00E-05 | 16 | 5 | 0.8716 | 0.7490 | 0.7450 |
| 9 | 5.00E-05 | 16 | 6 | 0.8348 | 0.6657 | 0.6750 |
| 9 | 5.00E-05 | 16 | 7 | 0.8188 | 0.6274 | 0.6400 |
| 9 | 5.00E-05 | 16 | 8 | 0.8242 | 0.6668 | 0.6750 |
| 9 | 5.00E-05 | 32 | 1 | 0.7745 | 0.6116 | 0.6250 |
| 9 | 5.00E-05 | 32 | 2 | 0.8365 | 0.6406 | 0.6650 |
| 9 | 5.00E-05 | 32 | 3 | 0.8290 | 0.6127 | 0.6400 |
| 9 | 5.00E-05 | 32 | 4 | 0.8513 | 0.7143 | 0.7150 |
| 9 | 5.00E-05 | 32 | 5 | 0.8433 | 0.6855 | 0.6850 |
| 9 | 5.00E-05 | 32 | 6 | 0.8272 | 0.6960 | 0.7000 |
| 9 | 5.00E-05 | 32 | 7 | 0.8190 | 0.6514 | 0.6550 |
| 9 | 5.00E-05 | 32 | 8 | 0.8366 | 0.7178 | 0.7250 |
| 10 | 2.00E-05 | 16 | 1 | 0.6852 | 0.4489 | 0.4800 |
| 10 | 2.00E-05 | 16 | 2 | 0.7798 | 0.6004 | 0.6050 |
| 10 | 2.00E-05 | 16 | 3 | 0.8260 | 0.6689 | 0.6750 |
| 10 | 2.00E-05 | 16 | 4 | 0.8125 | 0.6699 | 0.6750 |
| 10 | 2.00E-05 | 16 | 5 | 0.8204 | 0.6541 | 0.6600 |
| 10 | 2.00E-05 | 16 | 6 | 0.8141 | 0.6476 | 0.6500 |
| 10 | 2.00E-05 | 16 | 7 | 0.8171 | 0.6646 | 0.6650 |
| 10 | 2.00E-05 | 16 | 8 | 0.8325 | 0.6576 | 0.6600 |
| 10 | 2.00E-05 | 32 | 1 | 0.6818 | 0.4655 | 0.4950 |
| 10 | 2.00E-05 | 32 | 2 | 0.7106 | 0.5173 | 0.5350 |
| 10 | 2.00E-05 | 32 | 3 | 0.7760 | 0.6078 | 0.6200 |
| 10 | 2.00E-05 | 32 | 4 | 0.7885 | 0.5832 | 0.5850 |
| 10 | 2.00E-05 | 32 | 5 | 0.8090 | 0.6347 | 0.6400 |
| 10 | 2.00E-05 | 32 | 6 | 0.8019 | 0.6338 | 0.6350 |
| 10 | 2.00E-05 | 32 | 7 | 0.8058 | 0.6348 | 0.6350 |
| 10 | 2.00E-05 | 32 | 8 | 0.8048 | 0.6448 | 0.6500 |
| 10 | 5.00E-05 | 16 | 1 | 0.7261 | 0.5607 | 0.5750 |
| 10 | 5.00E-05 | 16 | 2 | 0.8480 | 0.7032 | 0.7050 |
| 10 | 5.00E-05 | 16 | 3 | 0.8741 | 0.7255 | 0.7300 |
| 10 | 5.00E-05 | 16 | 4 | 0.8805 | 0.7543 | 0.7550 |
| 10 | 5.00E-05 | 16 | 5 | 0.8458 | 0.7113 | 0.7150 |
| 10 | 5.00E-05 | 16 | 6 | 0.8479 | 0.7203 | 0.7250 |
| 10 | 5.00E-05 | 16 | 7 | 0.8324 | 0.6738 | 0.6800 |
| 10 | 5.00E-05 | 16 | 8 | 0.8655 | 0.6892 | 0.7000 |
| 10 | 5.00E-05 | 32 | 1 | 0.6919 | 0.4926 | 0.5150 |
| 10 | 5.00E-05 | 32 | 2 | 0.8085 | 0.6602 | 0.6600 |
| 10 | 5.00E-05 | 32 | 3 | 0.8726 | 0.7200 | 0.7200 |
| 10 | 5.00E-05 | 32 | 4 | 0.8269 | 0.6671 | 0.6650 |
| 10 | 5.00E-05 | 32 | 5 | 0.8093 | 0.6378 | 0.6400 |
| 10 | 5.00E-05 | 32 | 6 | 0.8233 | 0.6849 | 0.6900 |
| 10 | 5.00E-05 | 32 | 7 | 0.8277 | 0.6674 | 0.6800 |
| 10 | 5.00E-05 | 32 | 8 | 0.8653 | 0.7016 | 0.7000 |

**Supplementary Table 6: Subgroup Analysis and** **Test for Assumptions for Multivariable Regression Model**

Supplementary Table 6a: Subgroup Analysis

|  |  | **Asia (n=17)** |  | **Africa (n=13)** |  | **Americas (n=9)** |  | **Europe (n=7)** |  |
| --- | --- | --- | --- | --- | --- | --- | --- | --- | --- |
|  | **Treatment Topic** | **Beta (95% C.I. , Adjusted)** | ***Adjusted P-*value** | **Beta (95% C.I. , Adjusted)** | ***Adjusted P-*value** | **Beta (95% C.I. , Adjusted)** | ***Adjusted P-*value** | **Beta (95% C.I. , Adjusted)** | ***Adjusted P-*value** |
| **Overall Survival** | Chemotherapy | 0.182 [-0.561-0.925] | 1.00 | -0.017 [-0.759-0.724] | 1.000 | 0.458 [-0.458-1.375] | .35 | -0.787 [-1.766-0.191] | .11 |
|  | Enucleation | -1.518 [-2.602--0.434] | .007 | -0.812 [-1.412--0.021] | .04 | 0.399 [-0.495-1.293] | .43 | -0.234 [-1.255-0.787] | .94 |
| **Globe Salvage** | Chemotherapy | -0.399 [-1.034-0.236] | .27 | 0.17 [-0.902-1.243] | 1.00 | -0.531 [-2.873-1.812] | 1.00 | 1.674 [-4.198-7.545] | .75 |
|  | Enucleation | -0.672 [-1.751-0.407] | .28 | 0.470 [-1.459-2.399] | .99 | 0.424 [-1.815-2.662] | 1.00 | -1.032[-5.486-3.423] | .93 |

Supplementary Table 6a: Test for Assumptions

|  | **Type** | **Gross Topic** | Normality | Homoscedasticity | Independence | Rainbow test |
| --- | --- | --- | --- | --- | --- | --- |
| **Overall Survival** | Net Sentiment | All Tweets | 0.0705 | 0.6020 | 0.0829 | 0.9901 |
|  |  | Awareness | 0.4013 | 0.3945 | 0.0562 | 0.9696 |
|  |  | Patient Support | 0.0705 | 0.6465 | 0.0506 | 0.9532 |
|  |  | Presentation and Diagnosis | 0.2743 | 0.6038 | 0.0519 | 0.9849 |
|  |  | Treatment (All Form) | 0.3208 | 0.4302 | 0.0524 | 0.9643 |
|  | Strength of  Negative Sentiment | Treatment (Chemotherapy) | 0.0916 | 0.0939 | 0.3336 | 0.6598 |
|  |  | Treatment (Enucleation) | 0.1100 | 0.2496 | 0.3440 | 0.6978 |
| **Globe Salvage** | Net Sentiment | All Tweets | 0.4012 | 0.5281 | 0.2184 | 0.2889 |
|  |  | Awareness | 0.0805 | 0.8419 | 0.3386 | 0.3155 |
|  |  | Patient Support | 0.1542 | 0.9144 | 0.4295 | 0.1730 |
|  |  | Presentation and Diagnosis | 0.4626 | 0.1388 | 0.3058 | 0.4734 |
|  |  | Treatment (All Form) | 0.7721 | 0.8736 | 0.3370 | 0.1828 |
|  | Strength of  Negative Sentiment | Treatment (Chemotherapy) | 0.3930 | 0.9158 | 0.0549 | 0.5308 |
|  |  | Treatment (Enucleation) | 0.0916 | 0.0939 | 0.3336 | 0.6598 |

Normality was tested with Shapiro-Wilk test. Homoscedasticity was evaluated with Breusch-Pagan test. Independence was tested with Durbin-Watson test. Linearity was tested with Rainbow test.

**Supplementary Table 7: Overall Cross-validation Statistics for BERT Model for Patient Role Classification**

**Supplementary Table 7a: Summary of Cross-validation Statistics for Patient Role Classification**

| **Learning Rate** | **Batch Size** | **Epochs** | **AUC** | **F1 Score** | **Accuracy** |
| --- | --- | --- | --- | --- | --- |
| 2E-05 | 16 | 1 | 0.758 [0.738-0.779] | 0.694 [0.646-0.742] | 70.5% [67.0%-74.0%] |
| 2E-05 | 16 | 2 | 0.801 [0.781-0.822] | 0.734 [0.716-0.753] | 73.7% [71.9%-75.6%] |
| 2E-05 | 16 | 3 | 0.813 [0.793-0.834] | 0.735 [0.716-0.754] | 73.9% [72.2%-75.5%] |
| 2E-05 | 16 | 4 | 0.806 [0.786-0.825] | 0.730 [0.706-0.753] | 73.3% [71.0%-75.6%] |
| 2E-05 | 16 | 5 | 0.799 [0.786-0.813] | 0.730 [0.715-0.746] | 73.2% [71.7%-74.8%] |
| 2E-05 | 16 | 6 | 0.796 [0.775-0.818] | 0.727 [0.703-0.751] | 72.8% [70.5%-75.2%] |
| 2E-05 | 16 | 7 | 0.796 [0.777-0.814] | 0.722 [0.698-0.747] | 72.5% [70.2%-74.8%] |
| 2E-05 | 16 | 8 | 0.802 [0.787-0.817] | 0.729 [0.716-0.742] | 72.9% [71.6%-74.2%] |
| 2E-05 | 32 | 1 | 0.753 [0.728-0.778] | 0.689 [0.650-0.728] | 69.7% [66.4%-73.1%] |
| 2E-05 | 32 | 2 | 0.790 [0.772-0.808] | 0.732 [0.708-0.755] | 73.4% [71.1%-75.7%] |
| 2E-05 | 32 | 3 | 0.808 [0.789-0.826] | 0.734 [0.721-0.748] | 73.6% [72.3%-75.0%] |
| 2E-05 | 32 | 4 | 0.802 [0.787-0.818] | 0.728 [0.713-0.743] | 73.0% [71.6%-74.4%] |
| 2E-05 | 32 | 5 | 0.802 [0.777-0.827] | 0.737 [0.720-0.753] | 73.7% [72.1%-75.4%] |
| 2E-05 | 32 | 6 | 0.808 [0.786-0.830] | 0.730 [0.711-0.749] | 73.1% [71.2%-75.0%] |
| 2E-05 | 32 | 7 | 0.798 [0.780-0.817] | 0.733 [0.716-0.750] | 73.4% [71.7%-75.2%] |
| 2E-05 | 32 | 8 | 0.801 [0.789-0.812] | 0.726 [0.711-0.741] | 72.9% [71.6%-74.2%] |
| 5E-05 | 16 | 1 | 0.782 [0.750-0.814] | 0.712 [0.691-0.734] | 71.9% [70.1%-73.8%] |
| 5E-05 | 16 | 2 | 0.803 [0.787-0.820] | 0.727 [0.697-0.757] | 73.3% [70.7%-75.9%] |
| 5E-05 | 16 | 3 | 0.782 [0.758-0.806] | 0.703 [0.683-0.722] | 71.1% [69.5%-72.6%] |
| 5E-05 | 16 | 4 | 0.790 [0.770-0.810] | 0.732 [0.716-0.748] | 73.5% [72.0%-75.0%] |
| 5E-05 | 16 | 5 | 0.782 [0.763-0.800] | 0.717 [0.704-0.731] | 72.1% [70.7%-73.5%] |
| 5E-05 | 16 | 6 | 0.779 [0.762-0.796] | 0.726 [0.703-0.749] | 72.8% [70.5%-75.1%] |
| 5E-05 | 16 | 7 | 0.779 [0.759-0.799] | 0.720 [0.696-0.744] | 72.3% [69.8%-74.7%] |
| 5E-05 | 16 | 8 | 0.784 [0.765-0.802] | 0.728 [0.703-0.753] | 72.9% [70.5%-75.4%] |
| 5E-05 | 32 | 1 | 0.770 [0.753-0.787] | 0.691 [0.665-0.717] | 69.4% [66.8%-71.9%] |
| 5E-05 | 32 | 2 | 0.793 [0.762-0.824] | 0.733 [0.702-0.764] | 73.8% [70.8%-76.8%] |
| 5E-05 | 32 | 3 | 0.800 [0.780-0.820] | 0.731 [0.705-0.756] | 73.3% [70.8%-75.8%] |
| 5E-05 | 32 | 4 | 0.807 [0.790-0.824] | 0.737 [0.717-0.756] | 73.9% [72.0%-75.8%] |
| 5E-05 | 32 | 5 | 0.797 [0.778-0.817] | 0.732 [0.709-0.755] | 73.5% [71.2%-75.7%] |
| 5E-05 | 32 | 6 | 0.799 [0.782-0.816] | 0.732 [0.704-0.760] | 73.6% [71.2%-76.0%] |
| 5E-05 | 32 | 7 | 0.781 [0.765-0.798] | 0.714 [0.700-0.729] | 71.8% [70.4%-73.2%] |
| 5E-05 | 32 | 8 | 0.781 [0.749-0.813] | 0.699 [0.665-0.733] | 70.1% [66.7%-73.5%] |

**Supplementary Table 7b: Per-fold Performance of Cross-validation Statistics for Patient Role Classification**

| Fold | Learning Rate | Batch Size | Epochs | AUC | F1 Score | Accuracy |
| --- | --- | --- | --- | --- | --- | --- |
| 1 | 2.00E-05 | 16 | 1 | 0.7245 | 0.6840 | 0.6850 |
| 1 | 2.00E-05 | 16 | 2 | 0.7925 | 0.7342 | 0.7350 |
| 1 | 2.00E-05 | 16 | 3 | 0.7903 | 0.7307 | 0.7350 |
| 1 | 2.00E-05 | 16 | 4 | 0.7889 | 0.7355 | 0.7350 |
| 1 | 2.00E-05 | 16 | 5 | 0.7972 | 0.7345 | 0.7350 |
| 1 | 2.00E-05 | 16 | 6 | 0.7595 | 0.6633 | 0.6700 |
| 1 | 2.00E-05 | 16 | 7 | 0.7817 | 0.7293 | 0.7300 |
| 1 | 2.00E-05 | 16 | 8 | 0.7739 | 0.6956 | 0.6950 |
| 1 | 2.00E-05 | 32 | 1 | 0.7253 | 0.5601 | 0.6000 |
| 1 | 2.00E-05 | 32 | 2 | 0.7768 | 0.7390 | 0.7400 |
| 1 | 2.00E-05 | 32 | 3 | 0.7882 | 0.6993 | 0.7000 |
| 1 | 2.00E-05 | 32 | 4 | 0.7623 | 0.7151 | 0.7150 |
| 1 | 2.00E-05 | 32 | 5 | 0.7324 | 0.6900 | 0.6900 |
| 1 | 2.00E-05 | 32 | 6 | 0.7954 | 0.7281 | 0.7350 |
| 1 | 2.00E-05 | 32 | 7 | 0.8034 | 0.7735 | 0.7750 |
| 1 | 2.00E-05 | 32 | 8 | 0.7825 | 0.7306 | 0.7300 |
| 1 | 5.00E-05 | 16 | 1 | 0.7584 | 0.7252 | 0.7300 |
| 1 | 5.00E-05 | 16 | 2 | 0.7942 | 0.6747 | 0.6900 |
| 1 | 5.00E-05 | 16 | 3 | 0.7236 | 0.6844 | 0.6850 |
| 1 | 5.00E-05 | 16 | 4 | 0.7512 | 0.7054 | 0.7050 |
| 1 | 5.00E-05 | 16 | 5 | 0.7693 | 0.7149 | 0.7150 |
| 1 | 5.00E-05 | 16 | 6 | 0.7600 | 0.7036 | 0.7050 |
| 1 | 5.00E-05 | 16 | 7 | 0.7620 | 0.7206 | 0.7200 |
| 1 | 5.00E-05 | 16 | 8 | 0.7803 | 0.7197 | 0.7200 |
| 1 | 5.00E-05 | 32 | 1 | 0.7689 | 0.6939 | 0.7050 |
| 1 | 5.00E-05 | 32 | 2 | 0.7761 | 0.7576 | 0.7600 |
| 1 | 5.00E-05 | 32 | 3 | 0.8007 | 0.6988 | 0.7000 |
| 1 | 5.00E-05 | 32 | 4 | 0.8070 | 0.7555 | 0.7550 |
| 1 | 5.00E-05 | 32 | 5 | 0.7830 | 0.7252 | 0.7300 |
| 1 | 5.00E-05 | 32 | 6 | 0.7998 | 0.7550 | 0.7600 |
| 1 | 5.00E-05 | 32 | 7 | 0.7410 | 0.6997 | 0.7000 |
| 1 | 5.00E-05 | 32 | 8 | 0.7056 | 0.6409 | 0.6400 |
| 2 | 2.00E-05 | 16 | 1 | 0.8066 | 0.7821 | 0.7850 |
| 2 | 2.00E-05 | 16 | 2 | 0.8470 | 0.7754 | 0.7800 |
| 2 | 2.00E-05 | 16 | 3 | 0.8690 | 0.7884 | 0.7900 |
| 2 | 2.00E-05 | 16 | 4 | 0.8655 | 0.8134 | 0.8150 |
| 2 | 2.00E-05 | 16 | 5 | 0.8357 | 0.7553 | 0.7550 |
| 2 | 2.00E-05 | 16 | 6 | 0.8358 | 0.7555 | 0.7550 |
| 2 | 2.00E-05 | 16 | 7 | 0.8590 | 0.7904 | 0.7900 |
| 2 | 2.00E-05 | 16 | 8 | 0.8174 | 0.7255 | 0.7250 |
| 2 | 2.00E-05 | 32 | 1 | 0.7798 | 0.7279 | 0.7300 |
| 2 | 2.00E-05 | 32 | 2 | 0.8361 | 0.7802 | 0.7800 |
| 2 | 2.00E-05 | 32 | 3 | 0.8423 | 0.7253 | 0.7300 |
| 2 | 2.00E-05 | 32 | 4 | 0.8444 | 0.7606 | 0.7600 |
| 2 | 2.00E-05 | 32 | 5 | 0.8668 | 0.7849 | 0.7850 |
| 2 | 2.00E-05 | 32 | 6 | 0.8831 | 0.7855 | 0.7850 |
| 2 | 2.00E-05 | 32 | 7 | 0.8484 | 0.7495 | 0.7550 |
| 2 | 2.00E-05 | 32 | 8 | 0.8307 | 0.7602 | 0.7600 |
| 2 | 5.00E-05 | 16 | 1 | 0.8309 | 0.7393 | 0.7450 |
| 2 | 5.00E-05 | 16 | 2 | 0.8388 | 0.7995 | 0.8000 |
| 2 | 5.00E-05 | 16 | 3 | 0.8420 | 0.6765 | 0.7000 |
| 2 | 5.00E-05 | 16 | 4 | 0.8214 | 0.7606 | 0.7600 |
| 2 | 5.00E-05 | 16 | 5 | 0.8305 | 0.7179 | 0.7200 |
| 2 | 5.00E-05 | 16 | 6 | 0.8334 | 0.7778 | 0.7800 |
| 2 | 5.00E-05 | 16 | 7 | 0.8414 | 0.7905 | 0.7900 |
| 2 | 5.00E-05 | 16 | 8 | 0.8172 | 0.7704 | 0.7700 |
| 2 | 5.00E-05 | 32 | 1 | 0.8006 | 0.7327 | 0.7350 |
| 2 | 5.00E-05 | 32 | 2 | 0.8426 | 0.7975 | 0.8000 |
| 2 | 5.00E-05 | 32 | 3 | 0.8524 | 0.7927 | 0.7950 |
| 2 | 5.00E-05 | 32 | 4 | 0.8624 | 0.7706 | 0.7700 |
| 2 | 5.00E-05 | 32 | 5 | 0.8636 | 0.7970 | 0.8000 |
| 2 | 5.00E-05 | 32 | 6 | 0.8495 | 0.7798 | 0.7800 |
| 2 | 5.00E-05 | 32 | 7 | 0.8264 | 0.7468 | 0.7550 |
| 2 | 5.00E-05 | 32 | 8 | 0.8296 | 0.7402 | 0.7400 |
| 3 | 2.00E-05 | 16 | 1 | 0.7706 | 0.6840 | 0.6850 |
| 3 | 2.00E-05 | 16 | 2 | 0.8074 | 0.7431 | 0.7500 |
| 3 | 2.00E-05 | 16 | 3 | 0.8253 | 0.7517 | 0.7550 |
| 3 | 2.00E-05 | 16 | 4 | 0.8061 | 0.7296 | 0.7400 |
| 3 | 2.00E-05 | 16 | 5 | 0.8252 | 0.7104 | 0.7150 |
| 3 | 2.00E-05 | 16 | 6 | 0.7699 | 0.7041 | 0.7050 |
| 3 | 2.00E-05 | 16 | 7 | 0.7896 | 0.6789 | 0.6900 |
| 3 | 2.00E-05 | 16 | 8 | 0.7692 | 0.7155 | 0.7150 |
| 3 | 2.00E-05 | 32 | 1 | 0.8063 | 0.6851 | 0.6950 |
| 3 | 2.00E-05 | 32 | 2 | 0.8120 | 0.7245 | 0.7250 |
| 3 | 2.00E-05 | 32 | 3 | 0.8028 | 0.7356 | 0.7350 |
| 3 | 2.00E-05 | 32 | 4 | 0.8091 | 0.6939 | 0.7050 |
| 3 | 2.00E-05 | 32 | 5 | 0.8101 | 0.7407 | 0.7400 |
| 3 | 2.00E-05 | 32 | 6 | 0.7983 | 0.7349 | 0.7350 |
| 3 | 2.00E-05 | 32 | 7 | 0.7777 | 0.6993 | 0.7000 |
| 3 | 2.00E-05 | 32 | 8 | 0.8176 | 0.7380 | 0.7400 |
| 3 | 5.00E-05 | 16 | 1 | 0.7980 | 0.6924 | 0.6950 |
| 3 | 5.00E-05 | 16 | 2 | 0.8022 | 0.6633 | 0.6800 |
| 3 | 5.00E-05 | 16 | 3 | 0.7899 | 0.7179 | 0.7250 |
| 3 | 5.00E-05 | 16 | 4 | 0.8036 | 0.7071 | 0.7100 |
| 3 | 5.00E-05 | 16 | 5 | 0.7727 | 0.6984 | 0.7050 |
| 3 | 5.00E-05 | 16 | 6 | 0.7819 | 0.7078 | 0.7100 |
| 3 | 5.00E-05 | 16 | 7 | 0.7773 | 0.7271 | 0.7350 |
| 3 | 5.00E-05 | 16 | 8 | 0.7298 | 0.6993 | 0.7000 |
| 3 | 5.00E-05 | 32 | 1 | 0.7974 | 0.7256 | 0.7250 |
| 3 | 5.00E-05 | 32 | 2 | 0.8224 | 0.7503 | 0.7550 |
| 3 | 5.00E-05 | 32 | 3 | 0.8029 | 0.7189 | 0.7200 |
| 3 | 5.00E-05 | 32 | 4 | 0.8164 | 0.7104 | 0.7150 |
| 3 | 5.00E-05 | 32 | 5 | 0.8166 | 0.7602 | 0.7600 |
| 3 | 5.00E-05 | 32 | 6 | 0.7797 | 0.6462 | 0.6700 |
| 3 | 5.00E-05 | 32 | 7 | 0.7610 | 0.7193 | 0.7200 |
| 3 | 5.00E-05 | 32 | 8 | 0.8209 | 0.7385 | 0.7400 |
| 4 | 2.00E-05 | 16 | 1 | 0.7486 | 0.7394 | 0.7400 |
| 4 | 2.00E-05 | 16 | 2 | 0.7863 | 0.7357 | 0.7350 |
| 4 | 2.00E-05 | 16 | 3 | 0.8163 | 0.7197 | 0.7200 |
| 4 | 2.00E-05 | 16 | 4 | 0.7991 | 0.7254 | 0.7250 |
| 4 | 2.00E-05 | 16 | 5 | 0.7859 | 0.7523 | 0.7550 |
| 4 | 2.00E-05 | 16 | 6 | 0.8095 | 0.7542 | 0.7550 |
| 4 | 2.00E-05 | 16 | 7 | 0.7832 | 0.7249 | 0.7250 |
| 4 | 2.00E-05 | 16 | 8 | 0.8102 | 0.7297 | 0.7300 |
| 4 | 2.00E-05 | 32 | 1 | 0.7817 | 0.7393 | 0.7450 |
| 4 | 2.00E-05 | 32 | 2 | 0.7597 | 0.6768 | 0.6850 |
| 4 | 2.00E-05 | 32 | 3 | 0.7991 | 0.7374 | 0.7400 |
| 4 | 2.00E-05 | 32 | 4 | 0.7915 | 0.7207 | 0.7200 |
| 4 | 2.00E-05 | 32 | 5 | 0.8276 | 0.7456 | 0.7450 |
| 4 | 2.00E-05 | 32 | 6 | 0.8183 | 0.7455 | 0.7450 |
| 4 | 2.00E-05 | 32 | 7 | 0.8126 | 0.7422 | 0.7450 |
| 4 | 2.00E-05 | 32 | 8 | 0.8097 | 0.7357 | 0.7350 |
| 4 | 5.00E-05 | 16 | 1 | 0.7579 | 0.7051 | 0.7050 |
| 4 | 5.00E-05 | 16 | 2 | 0.7706 | 0.7155 | 0.7150 |
| 4 | 5.00E-05 | 16 | 3 | 0.7888 | 0.7100 | 0.7100 |
| 4 | 5.00E-05 | 16 | 4 | 0.8210 | 0.7542 | 0.7550 |
| 4 | 5.00E-05 | 16 | 5 | 0.7859 | 0.7433 | 0.7450 |
| 4 | 5.00E-05 | 16 | 6 | 0.8035 | 0.7604 | 0.7600 |
| 4 | 5.00E-05 | 16 | 7 | 0.7445 | 0.6956 | 0.6950 |
| 4 | 5.00E-05 | 16 | 8 | 0.8090 | 0.7057 | 0.7100 |
| 4 | 5.00E-05 | 32 | 1 | 0.7666 | 0.6705 | 0.6700 |
| 4 | 5.00E-05 | 32 | 2 | 0.7964 | 0.7281 | 0.7350 |
| 4 | 5.00E-05 | 32 | 3 | 0.8041 | 0.7475 | 0.7500 |
| 4 | 5.00E-05 | 32 | 4 | 0.8108 | 0.7451 | 0.7450 |
| 4 | 5.00E-05 | 32 | 5 | 0.8020 | 0.7449 | 0.7450 |
| 4 | 5.00E-05 | 32 | 6 | 0.7957 | 0.7289 | 0.7300 |
| 4 | 5.00E-05 | 32 | 7 | 0.7982 | 0.7267 | 0.7300 |
| 4 | 5.00E-05 | 32 | 8 | 0.8024 | 0.7490 | 0.7500 |
| 5 | 2.00E-05 | 16 | 1 | 0.7630 | 0.7192 | 0.7300 |
| 5 | 2.00E-05 | 16 | 2 | 0.8012 | 0.7649 | 0.7650 |
| 5 | 2.00E-05 | 16 | 3 | 0.8210 | 0.7351 | 0.7350 |
| 5 | 2.00E-05 | 16 | 4 | 0.7663 | 0.7271 | 0.7350 |
| 5 | 2.00E-05 | 16 | 5 | 0.7854 | 0.7580 | 0.7650 |
| 5 | 2.00E-05 | 16 | 6 | 0.7668 | 0.7254 | 0.7250 |
| 5 | 2.00E-05 | 16 | 7 | 0.7804 | 0.7238 | 0.7350 |
| 5 | 2.00E-05 | 16 | 8 | 0.7876 | 0.7455 | 0.7450 |
| 5 | 2.00E-05 | 32 | 1 | 0.7093 | 0.6455 | 0.6450 |
| 5 | 2.00E-05 | 32 | 2 | 0.7925 | 0.7307 | 0.7300 |
| 5 | 2.00E-05 | 32 | 3 | 0.7993 | 0.7400 | 0.7400 |
| 5 | 2.00E-05 | 32 | 4 | 0.8013 | 0.7422 | 0.7450 |
| 5 | 2.00E-05 | 32 | 5 | 0.7836 | 0.7206 | 0.7200 |
| 5 | 2.00E-05 | 32 | 6 | 0.7778 | 0.6997 | 0.7000 |
| 5 | 2.00E-05 | 32 | 7 | 0.7730 | 0.7361 | 0.7400 |
| 5 | 2.00E-05 | 32 | 8 | 0.7852 | 0.7107 | 0.7100 |
| 5 | 5.00E-05 | 16 | 1 | 0.8013 | 0.7089 | 0.7100 |
| 5 | 5.00E-05 | 16 | 2 | 0.7914 | 0.7700 | 0.7700 |
| 5 | 5.00E-05 | 16 | 3 | 0.7829 | 0.7254 | 0.7250 |
| 5 | 5.00E-05 | 16 | 4 | 0.7677 | 0.7138 | 0.7300 |
| 5 | 5.00E-05 | 16 | 5 | 0.7915 | 0.7180 | 0.7300 |
| 5 | 5.00E-05 | 16 | 6 | 0.7565 | 0.7477 | 0.7550 |
| 5 | 5.00E-05 | 16 | 7 | 0.7780 | 0.6954 | 0.6950 |
| 5 | 5.00E-05 | 16 | 8 | 0.8063 | 0.7840 | 0.7850 |
| 5 | 5.00E-05 | 32 | 1 | 0.7472 | 0.6708 | 0.6700 |
| 5 | 5.00E-05 | 32 | 2 | 0.7894 | 0.7066 | 0.7150 |
| 5 | 5.00E-05 | 32 | 3 | 0.7464 | 0.6692 | 0.6700 |
| 5 | 5.00E-05 | 32 | 4 | 0.7881 | 0.7290 | 0.7350 |
| 5 | 5.00E-05 | 32 | 5 | 0.7822 | 0.7148 | 0.7150 |
| 5 | 5.00E-05 | 32 | 6 | 0.7822 | 0.7254 | 0.7250 |
| 5 | 5.00E-05 | 32 | 7 | 0.7705 | 0.6802 | 0.7000 |
| 5 | 5.00E-05 | 32 | 8 | 0.8096 | 0.7084 | 0.7100 |
| 6 | 2.00E-05 | 16 | 1 | 0.7331 | 0.6970 | 0.7000 |
| 6 | 2.00E-05 | 16 | 2 | 0.7895 | 0.7244 | 0.7300 |
| 6 | 2.00E-05 | 16 | 3 | 0.8112 | 0.7385 | 0.7400 |
| 6 | 2.00E-05 | 16 | 4 | 0.7900 | 0.7154 | 0.7150 |
| 6 | 2.00E-05 | 16 | 5 | 0.7689 | 0.6988 | 0.7000 |
| 6 | 2.00E-05 | 16 | 6 | 0.8106 | 0.7477 | 0.7550 |
| 6 | 2.00E-05 | 16 | 7 | 0.7905 | 0.7054 | 0.7050 |
| 6 | 2.00E-05 | 16 | 8 | 0.7951 | 0.7106 | 0.7100 |
| 6 | 2.00E-05 | 32 | 1 | 0.7700 | 0.7158 | 0.7200 |
| 6 | 2.00E-05 | 32 | 2 | 0.7809 | 0.7524 | 0.7600 |
| 6 | 2.00E-05 | 32 | 3 | 0.7598 | 0.7401 | 0.7450 |
| 6 | 2.00E-05 | 32 | 4 | 0.8138 | 0.7404 | 0.7400 |
| 6 | 2.00E-05 | 32 | 5 | 0.7856 | 0.7314 | 0.7350 |
| 6 | 2.00E-05 | 32 | 6 | 0.7832 | 0.7226 | 0.7250 |
| 6 | 2.00E-05 | 32 | 7 | 0.7711 | 0.7107 | 0.7100 |
| 6 | 2.00E-05 | 32 | 8 | 0.8016 | 0.7307 | 0.7400 |
| 6 | 5.00E-05 | 16 | 1 | 0.7875 | 0.7043 | 0.7250 |
| 6 | 5.00E-05 | 16 | 2 | 0.8227 | 0.6998 | 0.7200 |
| 6 | 5.00E-05 | 16 | 3 | 0.7685 | 0.7327 | 0.7350 |
| 6 | 5.00E-05 | 16 | 4 | 0.7839 | 0.7415 | 0.7450 |
| 6 | 5.00E-05 | 16 | 5 | 0.7366 | 0.6852 | 0.6850 |
| 6 | 5.00E-05 | 16 | 6 | 0.7722 | 0.7307 | 0.7300 |
| 6 | 5.00E-05 | 16 | 7 | 0.8056 | 0.7273 | 0.7300 |
| 6 | 5.00E-05 | 16 | 8 | 0.7629 | 0.7232 | 0.7250 |
| 6 | 5.00E-05 | 32 | 1 | 0.7505 | 0.6658 | 0.6650 |
| 6 | 5.00E-05 | 32 | 2 | 0.7809 | 0.7142 | 0.7200 |
| 6 | 5.00E-05 | 32 | 3 | 0.8215 | 0.7456 | 0.7450 |
| 6 | 5.00E-05 | 32 | 4 | 0.7996 | 0.7713 | 0.7750 |
| 6 | 5.00E-05 | 32 | 5 | 0.7943 | 0.7421 | 0.7500 |
| 6 | 5.00E-05 | 32 | 6 | 0.8084 | 0.7486 | 0.7500 |
| 6 | 5.00E-05 | 32 | 7 | 0.7911 | 0.7337 | 0.7400 |
| 6 | 5.00E-05 | 32 | 8 | 0.7077 | 0.6155 | 0.6150 |
| 7 | 2.00E-05 | 16 | 1 | 0.7998 | 0.7590 | 0.7600 |
| 7 | 2.00E-05 | 16 | 2 | 0.8431 | 0.7281 | 0.7350 |
| 7 | 2.00E-05 | 16 | 3 | 0.8208 | 0.7237 | 0.7250 |
| 7 | 2.00E-05 | 16 | 4 | 0.7972 | 0.7281 | 0.7350 |
| 7 | 2.00E-05 | 16 | 5 | 0.7909 | 0.7400 | 0.7400 |
| 7 | 2.00E-05 | 16 | 6 | 0.8258 | 0.7600 | 0.7600 |
| 7 | 2.00E-05 | 16 | 7 | 0.8166 | 0.7556 | 0.7550 |
| 7 | 2.00E-05 | 16 | 8 | 0.8144 | 0.7505 | 0.7500 |
| 7 | 2.00E-05 | 32 | 1 | 0.7425 | 0.7328 | 0.7400 |
| 7 | 2.00E-05 | 32 | 2 | 0.8187 | 0.7571 | 0.7600 |
| 7 | 2.00E-05 | 32 | 3 | 0.8468 | 0.7586 | 0.7600 |
| 7 | 2.00E-05 | 32 | 4 | 0.8176 | 0.7481 | 0.7500 |
| 7 | 2.00E-05 | 32 | 5 | 0.8179 | 0.7380 | 0.7400 |
| 7 | 2.00E-05 | 32 | 6 | 0.8036 | 0.7428 | 0.7450 |
| 7 | 2.00E-05 | 32 | 7 | 0.8194 | 0.7446 | 0.7450 |
| 7 | 2.00E-05 | 32 | 8 | 0.7849 | 0.7380 | 0.7400 |
| 7 | 5.00E-05 | 16 | 1 | 0.8297 | 0.7510 | 0.7550 |
| 7 | 5.00E-05 | 16 | 2 | 0.8262 | 0.7463 | 0.7500 |
| 7 | 5.00E-05 | 16 | 3 | 0.7992 | 0.7271 | 0.7400 |
| 7 | 5.00E-05 | 16 | 4 | 0.8060 | 0.7646 | 0.7650 |
| 7 | 5.00E-05 | 16 | 5 | 0.7660 | 0.7204 | 0.7300 |
| 7 | 5.00E-05 | 16 | 6 | 0.7560 | 0.6658 | 0.6650 |
| 7 | 5.00E-05 | 16 | 7 | 0.7723 | 0.7075 | 0.7200 |
| 7 | 5.00E-05 | 16 | 8 | 0.8014 | 0.7653 | 0.7650 |
| 7 | 5.00E-05 | 32 | 1 | 0.7823 | 0.7250 | 0.7350 |
| 7 | 5.00E-05 | 32 | 2 | 0.8287 | 0.7576 | 0.7600 |
| 7 | 5.00E-05 | 32 | 3 | 0.8095 | 0.7594 | 0.7600 |
| 7 | 5.00E-05 | 32 | 4 | 0.7820 | 0.7300 | 0.7300 |
| 7 | 5.00E-05 | 32 | 5 | 0.7624 | 0.6911 | 0.7050 |
| 7 | 5.00E-05 | 32 | 6 | 0.8214 | 0.7791 | 0.7800 |
| 7 | 5.00E-05 | 32 | 7 | 0.7860 | 0.7294 | 0.7300 |
| 7 | 5.00E-05 | 32 | 8 | 0.7951 | 0.7256 | 0.7250 |
| 8 | 2.00E-05 | 16 | 1 | 0.7643 | 0.5402 | 0.6200 |
| 8 | 2.00E-05 | 16 | 2 | 0.8170 | 0.7449 | 0.7450 |
| 8 | 2.00E-05 | 16 | 3 | 0.8302 | 0.7504 | 0.7500 |
| 8 | 2.00E-05 | 16 | 4 | 0.8263 | 0.6817 | 0.6900 |
| 8 | 2.00E-05 | 16 | 5 | 0.8020 | 0.7304 | 0.7300 |
| 8 | 2.00E-05 | 16 | 6 | 0.7714 | 0.7093 | 0.7100 |
| 8 | 2.00E-05 | 16 | 7 | 0.7973 | 0.7342 | 0.7350 |
| 8 | 2.00E-05 | 16 | 8 | 0.8113 | 0.7314 | 0.7350 |
| 8 | 2.00E-05 | 32 | 1 | 0.7558 | 0.7169 | 0.7250 |
| 8 | 2.00E-05 | 32 | 2 | 0.7875 | 0.7571 | 0.7600 |
| 8 | 2.00E-05 | 32 | 3 | 0.8204 | 0.7393 | 0.7450 |
| 8 | 2.00E-05 | 32 | 4 | 0.7962 | 0.7097 | 0.7100 |
| 8 | 2.00E-05 | 32 | 5 | 0.8073 | 0.7408 | 0.7450 |
| 8 | 2.00E-05 | 32 | 6 | 0.7896 | 0.6988 | 0.7000 |
| 8 | 2.00E-05 | 32 | 7 | 0.7882 | 0.7306 | 0.7300 |
| 8 | 2.00E-05 | 32 | 8 | 0.7955 | 0.6836 | 0.7000 |
| 8 | 5.00E-05 | 16 | 1 | 0.8171 | 0.7364 | 0.7450 |
| 8 | 5.00E-05 | 16 | 2 | 0.8191 | 0.7506 | 0.7500 |
| 8 | 5.00E-05 | 16 | 3 | 0.7671 | 0.7072 | 0.7100 |
| 8 | 5.00E-05 | 16 | 4 | 0.7512 | 0.7197 | 0.7200 |
| 8 | 5.00E-05 | 16 | 5 | 0.8128 | 0.7494 | 0.7500 |
| 8 | 5.00E-05 | 16 | 6 | 0.7793 | 0.7337 | 0.7350 |
| 8 | 5.00E-05 | 16 | 7 | 0.7830 | 0.7422 | 0.7450 |
| 8 | 5.00E-05 | 16 | 8 | 0.7683 | 0.7332 | 0.7350 |
| 8 | 5.00E-05 | 32 | 1 | 0.7994 | 0.7207 | 0.7200 |
| 8 | 5.00E-05 | 32 | 2 | 0.8202 | 0.7644 | 0.7700 |
| 8 | 5.00E-05 | 32 | 3 | 0.8037 | 0.7448 | 0.7500 |
| 8 | 5.00E-05 | 32 | 4 | 0.7851 | 0.6823 | 0.6850 |
| 8 | 5.00E-05 | 32 | 5 | 0.7812 | 0.7345 | 0.7350 |
| 8 | 5.00E-05 | 32 | 6 | 0.7862 | 0.7397 | 0.7400 |
| 8 | 5.00E-05 | 32 | 7 | 0.7748 | 0.7041 | 0.7050 |
| 8 | 5.00E-05 | 32 | 8 | 0.8076 | 0.7279 | 0.7300 |
| 9 | 2.00E-05 | 16 | 1 | 0.7495 | 0.6802 | 0.6850 |
| 9 | 2.00E-05 | 16 | 2 | 0.7668 | 0.6956 | 0.6950 |
| 9 | 2.00E-05 | 16 | 3 | 0.7786 | 0.6855 | 0.7050 |
| 9 | 2.00E-05 | 16 | 4 | 0.7967 | 0.7151 | 0.7150 |
| 9 | 2.00E-05 | 16 | 5 | 0.7993 | 0.7238 | 0.7250 |
| 9 | 2.00E-05 | 16 | 6 | 0.7829 | 0.6906 | 0.6900 |
| 9 | 2.00E-05 | 16 | 7 | 0.7761 | 0.7056 | 0.7050 |
| 9 | 2.00E-05 | 16 | 8 | 0.8019 | 0.7424 | 0.7450 |
| 9 | 2.00E-05 | 32 | 1 | 0.6937 | 0.6635 | 0.6650 |
| 9 | 2.00E-05 | 32 | 2 | 0.7683 | 0.7049 | 0.7050 |
| 9 | 2.00E-05 | 32 | 3 | 0.7931 | 0.7100 | 0.7100 |
| 9 | 2.00E-05 | 32 | 4 | 0.7951 | 0.7376 | 0.7400 |
| 9 | 2.00E-05 | 32 | 5 | 0.7850 | 0.7400 | 0.7400 |
| 9 | 2.00E-05 | 32 | 6 | 0.7956 | 0.7002 | 0.7000 |
| 9 | 2.00E-05 | 32 | 7 | 0.7752 | 0.7004 | 0.7000 |
| 9 | 2.00E-05 | 32 | 8 | 0.8041 | 0.7055 | 0.7050 |
| 9 | 5.00E-05 | 16 | 1 | 0.6819 | 0.6451 | 0.6700 |
| 9 | 5.00E-05 | 16 | 2 | 0.7688 | 0.7097 | 0.7100 |
| 9 | 5.00E-05 | 16 | 3 | 0.7432 | 0.6472 | 0.6700 |
| 9 | 5.00E-05 | 16 | 4 | 0.7778 | 0.7247 | 0.7300 |
| 9 | 5.00E-05 | 16 | 5 | 0.7723 | 0.7228 | 0.7250 |
| 9 | 5.00E-05 | 16 | 6 | 0.7659 | 0.7059 | 0.7150 |
| 9 | 5.00E-05 | 16 | 7 | 0.7768 | 0.7323 | 0.7350 |
| 9 | 5.00E-05 | 16 | 8 | 0.7804 | 0.6793 | 0.6800 |
| 9 | 5.00E-05 | 32 | 1 | 0.7538 | 0.6836 | 0.6850 |
| 9 | 5.00E-05 | 32 | 2 | 0.6894 | 0.6363 | 0.6450 |
| 9 | 5.00E-05 | 32 | 3 | 0.7738 | 0.6977 | 0.7050 |
| 9 | 5.00E-05 | 32 | 4 | 0.7925 | 0.7482 | 0.7500 |
| 9 | 5.00E-05 | 32 | 5 | 0.7974 | 0.6906 | 0.6900 |
| 9 | 5.00E-05 | 32 | 6 | 0.7677 | 0.7024 | 0.7100 |
| 9 | 5.00E-05 | 32 | 7 | 0.7729 | 0.7055 | 0.7050 |
| 9 | 5.00E-05 | 32 | 8 | 0.7546 | 0.6482 | 0.6500 |
| 10 | 2.00E-05 | 16 | 1 | 0.7220 | 0.6513 | 0.6583 |
| 10 | 2.00E-05 | 16 | 2 | 0.7641 | 0.6979 | 0.7035 |
| 10 | 2.00E-05 | 16 | 3 | 0.7717 | 0.7294 | 0.7337 |
| 10 | 2.00E-05 | 16 | 4 | 0.8199 | 0.7271 | 0.7286 |
| 10 | 2.00E-05 | 16 | 5 | 0.8038 | 0.7007 | 0.7035 |
| 10 | 2.00E-05 | 16 | 6 | 0.8321 | 0.7589 | 0.7588 |
| 10 | 2.00E-05 | 16 | 7 | 0.7806 | 0.6763 | 0.6784 |
| 10 | 2.00E-05 | 16 | 8 | 0.8398 | 0.7443 | 0.7437 |
| 10 | 2.00E-05 | 32 | 1 | 0.7647 | 0.7035 | 0.7085 |
| 10 | 2.00E-05 | 32 | 2 | 0.7660 | 0.6931 | 0.6935 |
| 10 | 2.00E-05 | 32 | 3 | 0.8241 | 0.7585 | 0.7588 |
| 10 | 2.00E-05 | 32 | 4 | 0.7904 | 0.7139 | 0.7136 |
| 10 | 2.00E-05 | 32 | 5 | 0.8012 | 0.7340 | 0.7337 |
| 10 | 2.00E-05 | 32 | 6 | 0.8313 | 0.7381 | 0.7387 |
| 10 | 2.00E-05 | 32 | 7 | 0.8138 | 0.7425 | 0.7437 |
| 10 | 2.00E-05 | 32 | 8 | 0.7935 | 0.7286 | 0.7286 |
| 10 | 5.00E-05 | 16 | 1 | 0.7604 | 0.7142 | 0.7136 |
| 10 | 5.00E-05 | 16 | 2 | 0.8000 | 0.7415 | 0.7437 |
| 10 | 5.00E-05 | 16 | 3 | 0.8133 | 0.6970 | 0.7085 |
| 10 | 5.00E-05 | 16 | 4 | 0.8170 | 0.7280 | 0.7286 |
| 10 | 5.00E-05 | 16 | 5 | 0.7781 | 0.7042 | 0.7035 |
| 10 | 5.00E-05 | 16 | 6 | 0.7805 | 0.7237 | 0.7236 |
| 10 | 5.00E-05 | 16 | 7 | 0.7477 | 0.6615 | 0.6633 |
| 10 | 5.00E-05 | 16 | 8 | 0.7804 | 0.7010 | 0.7035 |
| 10 | 5.00E-05 | 32 | 1 | 0.7333 | 0.6189 | 0.6281 |
| 10 | 5.00E-05 | 32 | 2 | 0.7835 | 0.7170 | 0.7186 |
| 10 | 5.00E-05 | 32 | 3 | 0.7845 | 0.7335 | 0.7337 |
| 10 | 5.00E-05 | 32 | 4 | 0.8248 | 0.7260 | 0.7286 |
| 10 | 5.00E-05 | 32 | 5 | 0.7913 | 0.7170 | 0.7186 |
| 10 | 5.00E-05 | 32 | 6 | 0.8002 | 0.7170 | 0.7186 |
| 10 | 5.00E-05 | 32 | 7 | 0.7906 | 0.6976 | 0.6985 |
| 10 | 5.00E-05 | 32 | 8 | 0.7780 | 0.7006 | 0.7085 |

Supplementary Table 8: Sentiment Comparison By Patient Role and Topic

|  | Patient and Relatives | Other Users | Adjusted P-value |
| --- | --- | --- | --- |
| Overall | -0.124 | 0.160 | <0.0001 |
| Awareness | -0.175 | 0.139 | <0.0001 |
| Other | -0.059 | 0.143 | <0.0001 |
| Patient Support | -0.148 | 0.310 | <0.0001 |
| Presentation and Diagnosis | -0.071 | 0.084 | <0.0001 |
| Treatment | -0.197 | 0.242 | <0.0001 |

P-value adjusted by Bonferroni method.

**Supplementary Table 9: Keywords Identified by Hierarchical Dirichlet Process on Reasons of Enucleation Hesitancy**

Topics and respective keywords were generated with unsupervised Hierarchical Dirichlet Process (HDP) model. We fused similar topics together and summarised the major areas, subtopics, and topic keywords for each platform. Example tweets were not included since only aggregate data can be presented according to privacy agreement.

| ID | Domains in Health Belief Model | Topic | Keywords |
| --- | --- | --- | --- |
| 0 | Perceived susceptibility | Denial of diagnosis | Wrong think wait lie diagnosis |
| 1 |  | Incidental Finding | Found brain exam spread scan |
| 2 | Perceived severity | Lack of Disease Understanding | Mystery find source confused challenge |
| 3 |  | Parent Overwhelmed by Diagnosis | Cry fear pain urgent perspective |
| 4 | Perceived benefits | Opt for Alternative Therapy | Research prevent blind expert believe |
| 5 |  | Losing Vision | Brother care blind retinoblastoma help |
| 6 |  | Worry of Surgery Failure and Complications | Lose infection concern death failure |
| 7 |  | Worry of Insufficient Control | Concern risk spread keep stop |
| 8 | Perceived barriers | Peer Pressure and Bullying | School disrespect attack bully friend |
| 9 |  | Gender discrimination for Girls | Girl threaten discrimination feminist social |
| 10 |  | Reduction in Government Support | Fund cut answer insurance policy |
| 11 |  | Require Support for Treatment | Help costly treatment need fund |
