# Supplementary material for: A Machine Learning Pipeline to Analyze Global Sentiment and Factors Influencing Retinoblastoma Treatment Hesitancy: Observational Infodemiology Study
Source: J Med Internet Res. 2026 Aug 11;28:e73364. doi: 10.2196/73364 (PMC13460677; doi:10.2196/73364)
Supplement: Supplementary file 2 [file jmir-v28-e73364-s002.docx]

**Supplementary Figure 1: Schematic Diagram Showing Dataflow for Analysis**

The flowchart shows the data flow of preprocessing and analysis for the current study.

**Supplementary Figure 2: Global Distribution of Twitter Tweets**

The choropleth map visualized the number of Twitter users sampled by location in our current study. The color scale was in log scale.

Supplementary Figure 3: Topic Distribution for Twitter

Supplementary Figure 4: BERTopic Topic Modelling

Supplementary Figure 4a: Topic Distribution by BERTopic model

Supplementary Figure 4b: Hierarchical Topic Clustering by BERTopic model

**Supplementary Figure 5: Engagement Statistics by Author Occupation**

Figures show the density distribution for passive response (i.e., likes) and active response (i.e., quoting, retweeting, replying), stratified by author’s occupation.

Supplementary Figure 5a: Passive Response Frequency for Posts by Author Occupation

Supplementary Figure 5b: Active Response Frequency for Posts by Author Occupation

These plots demonstrate the active and passive interaction count for posts from different professions. For each sub-plot, the upper density plot shows the proportion of users at different interaction frequency on log scale. The lower plot highlights the concentration of users at different response frequency, with darker bands showing higher frequency.

**Supplementary Figure 6: Plot Showing Network Plot Global Network of Retinoblastoma Information Flow on Twitter**

The participation of specialized users, i.e., not general users, was limited, as evidenced by network analysis. The global network consisted of 797,870 nodes and 826328 edges. The average degree was 0.8411. The network diameter was 11, and the average path length was 4.5748. For user stratification, general users accounted for 83.87% of users on the global network. Media outlets and creative media creators accounted for 11.18% of users, clinical medicine practitioners 1.80%, and educators/ academia 1.86%. Politicians and participants from public administration accounted for 1.17% of users, while 0.12% of users engaged in religious service.

The global network for the top 10000 users with specific occupations by eigenvector centrality was visualized in this diagram. Users with clinical backgrounds were concentrated in Northern America and Western Europe, with frequent information exchange. Media outlets and creative media creators in the above regions also served as hubs for information dissemination to the rest of the world, where users also relayed information locally. Politicians and public administrators in North America also showed a high eigenvector centrality, contributing notably to spreading information. Occasional regional transactions were observed between parts of South America and Africa, as well as Africa and Southern Asia.
